# Traditional utilization, botany, phytochemistry, pharmacology, pharmaceutical analysis, processing and application of the seeds of *Herpetospermum pedunculosum* (Ser.) C.B. Clarke: a comprehensive review

**DOI:** 10.3389/fphar.2024.1498768

**Published:** 2024-12-19

**Authors:** Zhixia Jiang, Chuang Zhang, Xinran Yu, Kaiyi Wang, Zhenqi Sang, Wan Gong, Qiaoyan Zhang, Xiongyu Meng, Lupin Qin, Qiming Zhao

**Affiliations:** College of Pharmaceutical Sciences, Zhejiang Chinese Medical University, Hangzhou, China

**Keywords:** *Herpetospermum pedunculosum* (Ser.) C.B. Clarke, phytochemistry, pharmacological activity, liver protection, lignan

## Abstract

The seed of *Herpetospermum pedunculosum* (Ser.) C.B. Clarke, known in Chinese as Bo-Leng-Gua-Zi and in Tibetan as Sejimedo, are here abbreviated as *H. pedunculosum* seeds. *Herpetospermum pedunculosum* seeds is a traditional Chinese medicine for protecting the liver, clearing heat, and detoxifying. A total of 125 chemical metabolites of *H. pedunculosum* seeds are found, including lignans, fatty acids, terpenes, coumarins, and others. The pharmacological activities of *H. pedunculosum* seeds are mainly in hepatoprotective, antioxidant, anti-cancer cells, and anticholestatic effects. In clinical application, it is mainly used in combination with other traditional Chinese medicines to play a key role in treating the liver disease. This paper gives a systematic review of above research aspects, proposes the potential limitations and put forward plausible solutions. Relevant literatures were searched in PubMed, Web of Science and Chinese National Knowledge Infrastructure with *Herpetospermum* as the key word. A number of studies have shown that *H. pedunculosum* seeds exert excellent hepatoprotective effects by acting on NF-κB, TGF-β, and Keap1-Nrf2 signaling pathways, which provide a solid base for its clinic application. However, more research is needed to explore the standard cultivation and quality evaluation of *H. pedunculosum* seeds and systematical structure-activity relationship of its active metabolites.

## 1 Introduction

With the continuous development of various drugs, phytomedicines with fewer side effects and significant effects are gradually attracting people. Especially for some effective ethnodrugs, further development of their hidden medicinal value through in-depth research is increasingly popular and desirable. *Herpetospermum pedunculosum* (Ser.) C.B. Clarke (*H. pedunculosum*) is mainly distributed in several high-altitude areas such as Tibet, Yunnan, India and Nepal. As the main medicinal part of *H. pedunculosum* (Ser.) C.B. Clarke, *H. pedunculosum* seeds are traditional Tibetan drug, which have traditional effects of clearing heat and softening liver. At the same time, *H. pedunculosum* seeds are also the core ingredient of clinical traditional Chinese prescriptions, such as Jiuwei Zhangya Pill (for treating cholecystitis), Wuwei Jinse Pill (for treating jaundice hepatitis), and Songshi pill (for treating hepatitis and liver fibrosis). Modern studies have shown that *H. pedunculosum* seeds contain a variety of chemical metabolites, including lignans, coumarins, terpenes, etc. (Xu, 2012), and have shown a variety of pharmacological activities, including liver protection, antioxidant, anti-tumor, and anti-cholestasis effects (Gong, 2012; Shen et al., 2015).

The excellent pharmacological effects and particular characteristics of *H. pedunculosum* seeds undoubtedly deserve systematical induction and summary, which is hardly reported to the best of our knowledge. Therefore, we investigated relevant literatures in Web of Science, PubMed and CNKI with Herpetospermum as the key word, and focused on the literatures of the *H*. *pedunculosum* seeds with excluding that on other parts, such as stem, leaf and flesh. Based on these literatures, this paper comprehensively reviews the traditional use, botany, chemical metabolites, pharmacological effects, pharmaceutical analysis, processing and application in Chinese herbal prescriptions of *H. pedunculosum* seeds, which can provide scientific basis for further research and promote the potential for development.

## 2 Traditional uses

As a classic Tibetan medicine, *H. pedunculosum* seeds often used in the treatment of Tri-pa (a disease be traditionally characterized by diffusion of bile, disorders of the blood-heat, and yellow color in the muscles and eyes), which was first recorded in Yue Wang Yao Zhen (《月王药诊》) in the early 8th century. At the same time, in the middle of the 8th century, Tara Materia Medica (《度母本草》, Shivatso) recorded that *H. pedunculosum* seeds can treat heat disease, and bacon disease (diseases caused by the combination of food accumulation and cold). Beside these, Si Bu Yi Dian (《四部医典》, Yutog Yontan Gonpo), written and revised during the late 8th to 12th century, further proposed that *H. pedunculosum* seeds can remove the heat of the lower organs. In addition, it supplemented the bitter taste of *H. pedunculosum* seeds. Jingzhu Materia Medica (《晶珠本草》, Dema Tenpe Nyima), written in 1840, proposed that *H. pedunculosum* seeds could treat Tri-pa in the viscera. Diqing Tibetan medicine (《迪庆藏药》, Yang and Chu cheng) and Chinese Tibetan medicine (《中华藏本草》Luo, 1997) supplement recorded its effects of treating liver and gallbladder heat and indigestion, which was also supported by the record of Chinese Materia Medica (《中华本草》, National Administration of Traditional Chinese Medicine). In 2015, the “Interpretation of Tibetan Medicine Jinsui Materia Medica” (《藏药金穗本草诠释》, Gama Qupei) concluded that *H. pedunculosum* seeds could treat the liver and gallbladder diseases of the Tri-pa type. In summary, *H. pedunculosum* seeds have been used as its prototype medicine for over 13 centuries, and its effects on protecting the liver and treating indigestion have gained tremendous application as recorded in traditional medical books.

## 3 Botany

**FIGURE 1:**
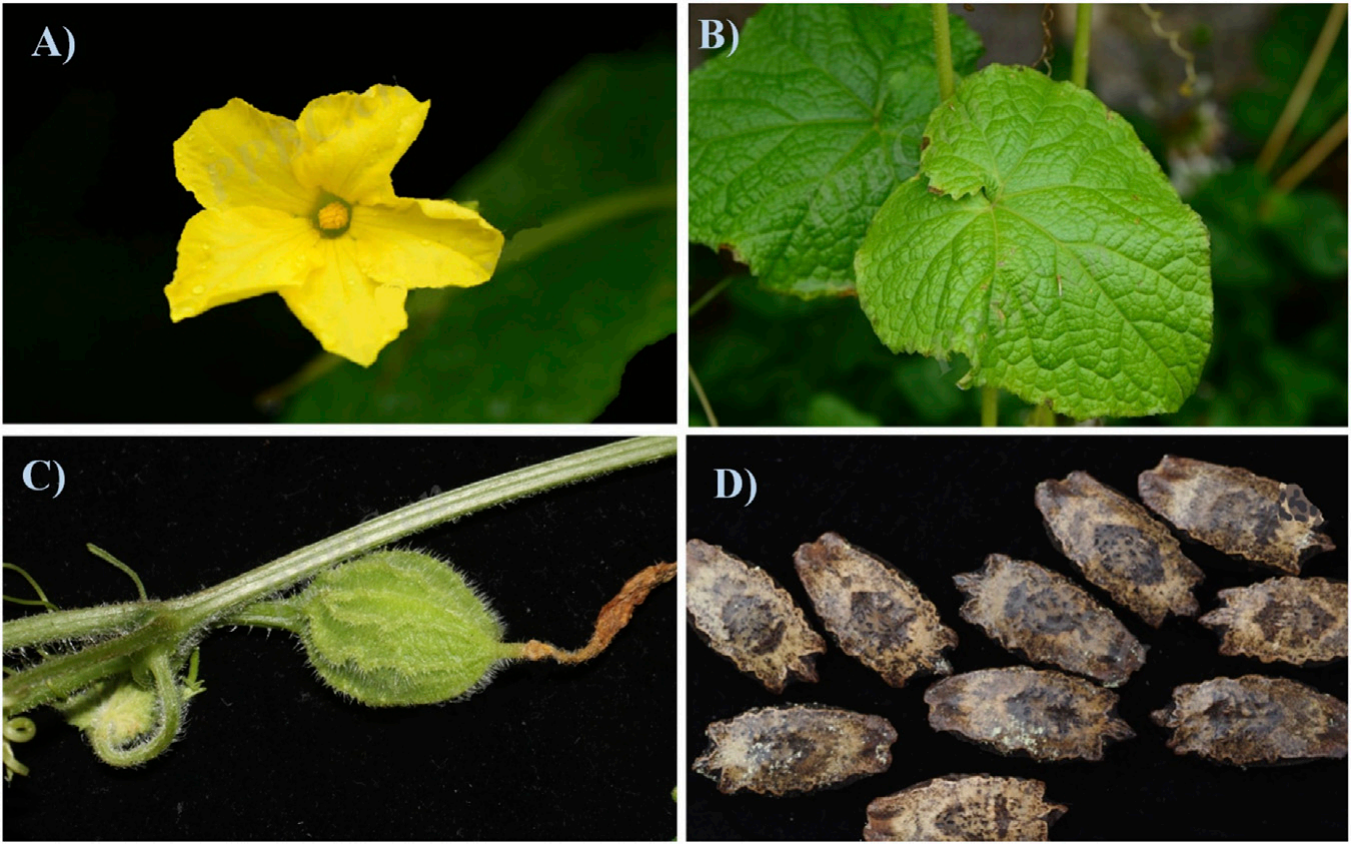
Flower **(A)**, leaf **(B)**, fruit **(C)** and seed **(D)** of *Herpetospermum pedunculosum* (Ser.) C.B. Clarke.

*Herpetospermum pedunculosum* (Ser.) C. B. Clarke, is usually harvested at around October, and adapts to grow on warm, humid subtropical roadsides, hillsides, shrubland, and forest edges at the altitude of 2,300–3,500 m (Flora Reipublicae Popularis Sinicae Commission, 1983) and its botanical organs including the flower, leaf, fruit and seed were shown in Figures 1A–D. As displayed in Figure 1D, *H. pedunculosum* seeds is slightly oblong with uneven carving and the surface from brown to black brown. One end of *H. pedunculosum* seeds has triangular protrusions, and the other end is tapered, slightly wedge-shaped and slightly concave in the center (Chinese Materia Medica Commission, 1998). The further investigation of *H. pedunculosum* seeds characters and sources can enhance the standardization of commercial *H. pedunculosum* seeds and is of great significance in cultivating it.

## 4 Phytochemistry

**FIGURE 2:**
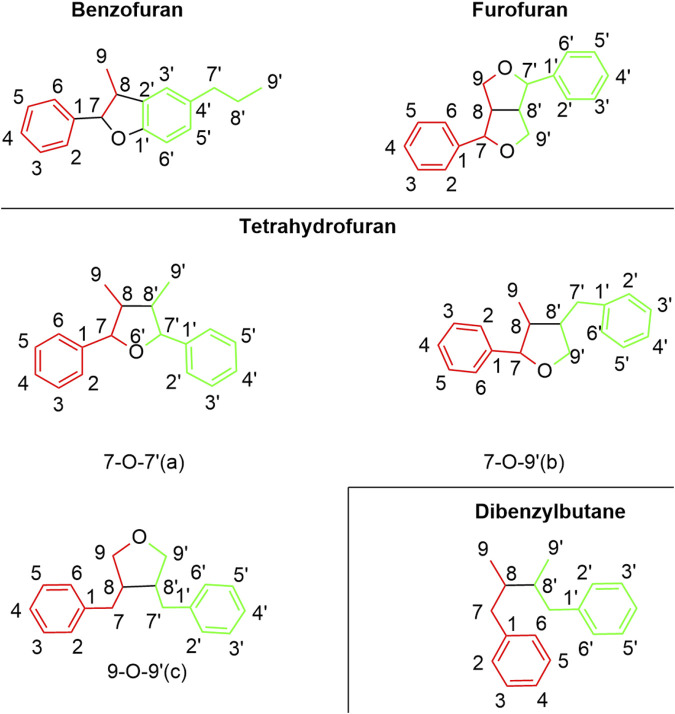
Lignan types in *Herpetospermum pedunculosum* seeds.

**FIGURE 3:**
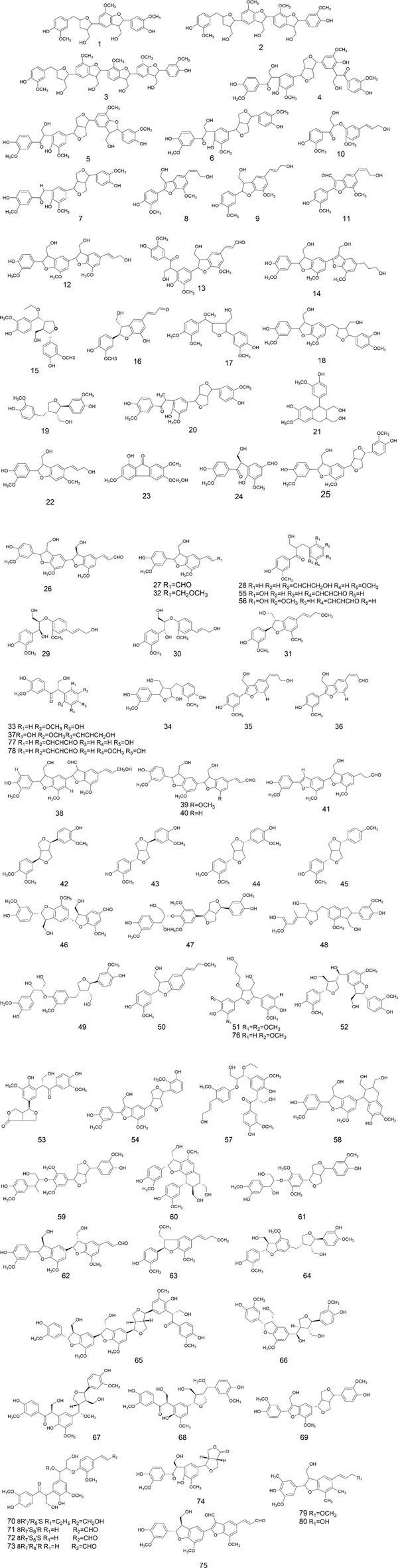
Structures of lignan metabolites in *Herpetospermum pedunculosum* seeds.

**FIGURE 4:**
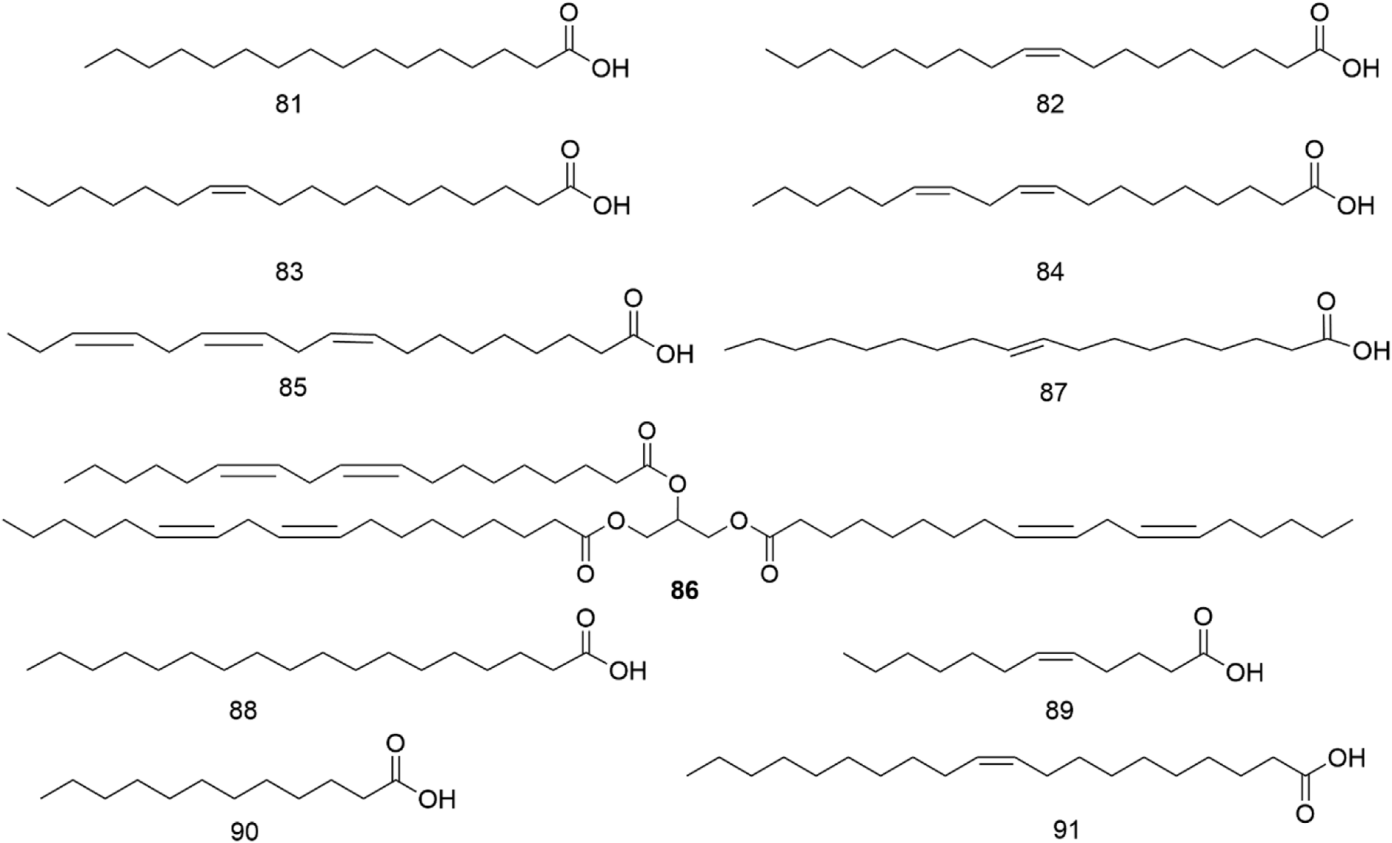
Structures of fatty acids from *Herpetospermum pedunculosum* seeds.

**FIGURE 5:**
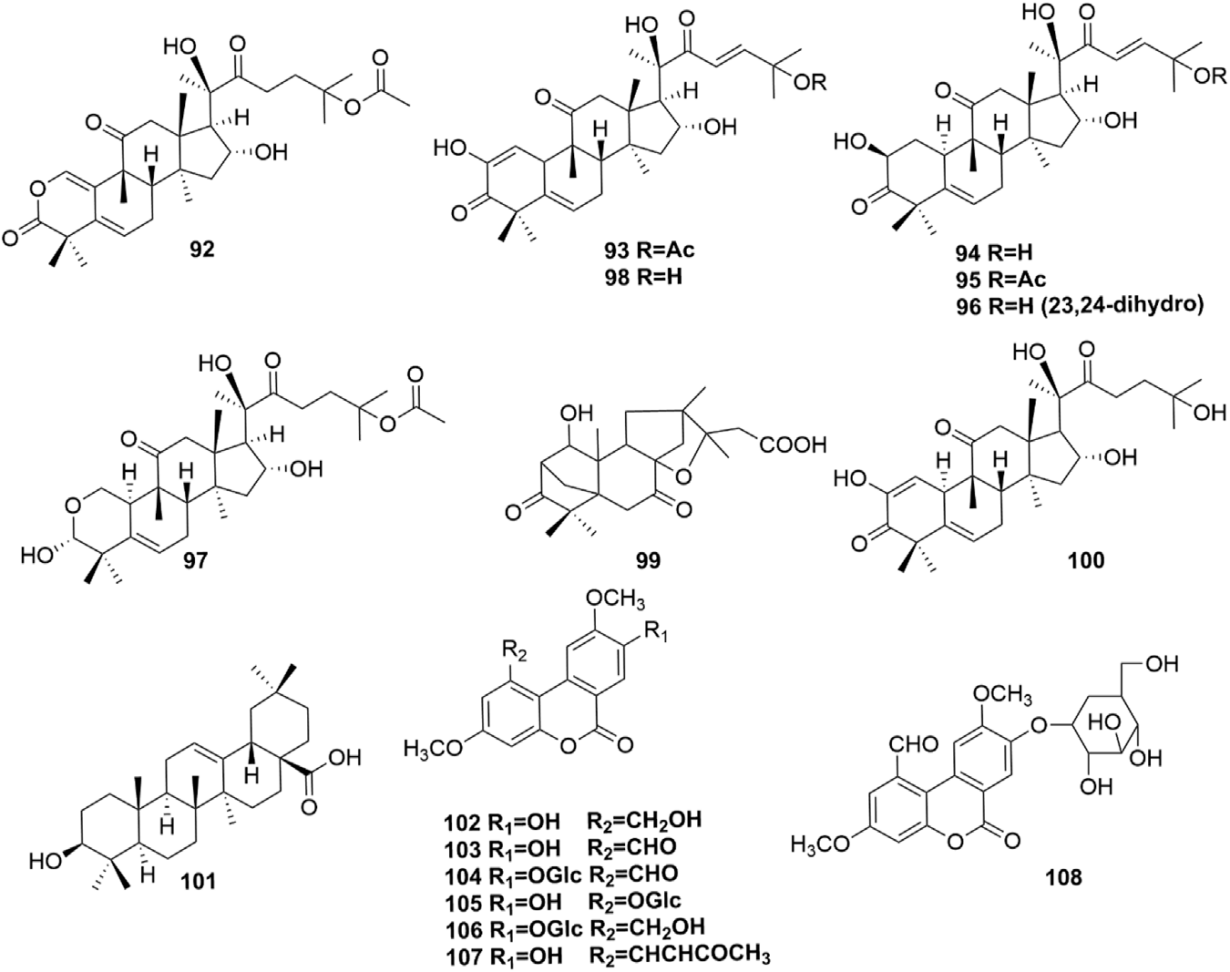
Structures of terpenoids (92–101) and coumarins (102–108).

**FIGURE 6:**
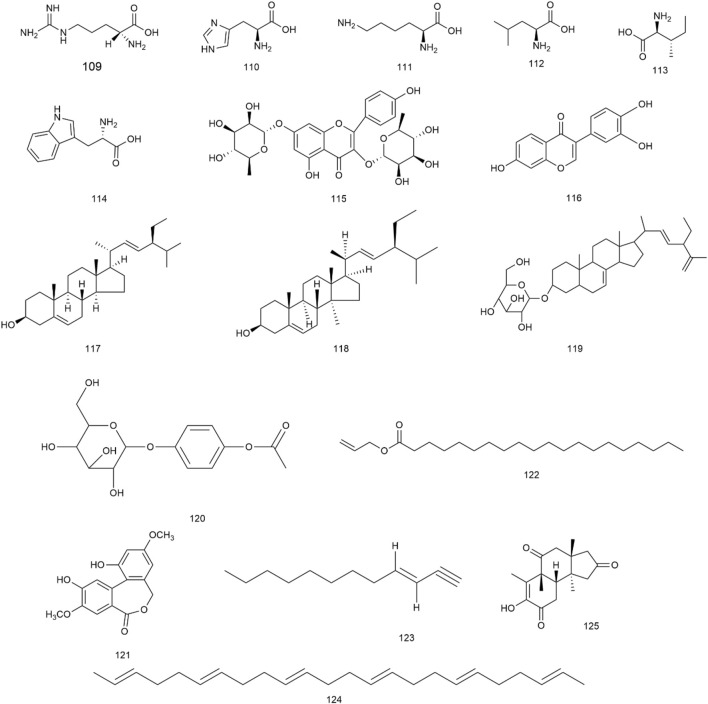
Structures of other metabolites from *Herpetospermum pedunculosum* seeds.

The chemical metabolites of *H. pedunculosum* seeds are reported to include lignans, fatty acids, terpenoids, and coumarins. The other metabolites such as amino acids, alkaloids, and flavones were also discussed. Details can be found in Figures 2–6 and Table 1.

**TABLE 1:** Metabolites extracted from *Herpetospermum pedunculosum* seeds.

No.	Name	Molecular formula	Extract	Separation method	Identification method	References
Lignan						
1	Herpetriol	C_30_H_34_O_9_	Ethyl alcohol	—	UV, IR,MS, ^1^H-NMR, ^13^C-NMR	Kaouadji et al. (1979)
2	Herpetetrol	C_40_H_44_O_12_	Ethyl alcohol	—	UV, MS, ^1^H-NMR, ^13^C-NMR	Kaouadji et al. (1979)
3	Herpepentol	C_50_H_54_O_15_	MeOH	—	MS, ^1^H-NMR, ^13^C-NMR	Kaouadji and Pieraccini (1984a)
4	Herpetetradione	C_40_H_42_O_12_	MeOH	—	MS, ^1^H-NMR, ^13^C-NMR	Kaouadji and Favre-Bonvin (1984a)
5	Herpetetrone	C_40_H_42_O_13_	MeOH	Polyamide CC6 and sephadex LH-20, chromatography	UV, IR, MS, ^1^H-NMR	Kaouadji et al. (1987)
6	Herpetrione	C_30_H_32_O_10_	Ethyl alcohol	—	MS, ^1^H-NMR, ^13^C-NMR	Kaouadji and Jean (1983)
7	Herpetone	C_29_H_30_O_9_	Ethyl alcohol	Silica gel column chromatography, preparative HPLC	MS, IR, ^1^H-NMR, ^13^C-NMR	Zhang et al. (2006)
8	Herpetol	C_20_H_20_O_6_	Ethyl alcohol	—	UV, MS, ^1^H-NMR, ^13^C-NMR	Kaouadji and Pieraccini (1984a)
9	Dehydrodiconiferyl alcohol	C_20_H_22_O_6_	Ethyl acetate	Normal phase silica gel column chromatography, MPLC, semi-preparative HPLC	UV, ^1^H-NMR, ^13^C-NMR	Ma (2020)
10	Herpetosin B	C_20_H_22_O_7_	Ethyl acetate	Silica gel column chromatography	UV, IR, MS, ^13^C-NMR, ^1^H-NMR	Xu (2012)
11	Herpetal	C_20_H_18_O_6_	Ethyl acetate	—	UV, ^1^H-NMR, ^13^C-NMR	Kaouadji et al. (1978)
12	Herpetotriol	C_30_H_32_O_9_	Ethyl acetate	—	UV, ^1^H-NMR, ^13^C-NMR	Kaouadji et al. (1978)
13	Herpepropenal	C_30_H_30_O_10_	Ethyl acetate	Silica gel column chromatography, RPC18, HPLC	MS, ^13^C-NMR, ^1^H-NMR, DEPT, HMBC, COSY, HSQC, NOESY	Yang et al. (2010)
14	7,8′-didehydlroherpetotriol	C_30_H_32_O_9_	Ethyl acetate	Reversed phase silica gel column chromatography, Preparation for HPLC Chromatography	UV, IR, MS, ^1^H-NMR,^13^C-NMR	Xu (2012)
15	(7S,8R,7′R,8′S)-7'-[7′-ethoxyl-7'-(4′-hydroxyl-3′-methoxylphenyl)]methyl-7-(4-hydroxyl-3-methoxylphenyl)-8-hydroxymethyl-tetrahydrofuran	C_22_H_28_O_7_	Ethyl acetate	Normal phase silica gel column chromatography, MPLC, semi- preparative HPLC	UV, IR, ^1^H-NMR, ^13^C-NMR	Ma (2020)
16	(7S,8R)-threo-1'-[3′-hydroxy-7-(4-hydroxy-3-methoxyphenyl)-8-hydroxymethyl-7,8-dihydrobenzofuran] acrylaldehyde	C_19_H_18_O_6_	Ethyl aceta	Normal phase silica gel column chromatography, MPLC, semi- preparative HPLC	UV, IR, ^1^H-NMR, ^13^C-NMR	Ma (2020)
17	(−)-Tanegool-7′-methyl etherl	C_21_H_26_O_7_	Ethyl acetate	Silica gel column chromatography, sephadex LH-20	MS, ^1^H-NMR, ^13^C-NMR	Zhou (2014)
18	Herpetin	C_30_H_34_O_9_	Ethyl acetate	Silica gel column chromatography, Rp-Si -gel, semi- preparative HPLC	MS, IR, ^1^H-NMR, ^13^C-NMR	Yuan et al. (2005)
19	Lariciresino	C_20_H_24_O_6_	Ethyl acetate	Normal phase silica gel column chromatography, MPLC, semi-preparative HPLC	UV, ^1^H-NMR,^13^C-NMR	Ma (2020)
20	(+)-(7′S,7′′S,8′R,8′′R)-4,4′,4′′-Trihydroxy-3,5′,3′′-trimethoxy-7-oxo-8-ene[8-3′,7′-O-9′′,8′-8′′,9′-O-7′′] lignoid	C_30_H_30_O_9_	Petroleum ether	Silica gel column chromatography, preparative HPLC	MS, IR, ^1^H-NMR, ^13^C-NMR, 1H-1H COSY, HMQC, HMBC	Yu et al. (2014)
21	Ent-isolariciresinol	C_20_H_24_O_6_	Ethyl acetate	Silica gel column chromatography, MPLC, semi-preparative HPLC	UV, MS, ^1^H-NMR, ^13^C-NMR	Ma (2020)
22	Herpetenol	C_20_H_22_O_6_	Ethyl acetate	Silica gel column chromatography	UV, IR, MS, ^1^H-NMR, ^13^C-NMR	Wang (2005)
23	Herpetfluorenone	C_16_H_14_O_6_	Ethyl acetate	Silica gel column chromatography, sephadex LH-20	MS, ^1^H-NMR, ^13^C-NMR	Gong et al. (2016)
24	(1S)-4hydroxy-3-[2-(4-hydroxy-3-methoxy-phenyl)-1-hydroxymethyl2-oxo-ethyl]-5-methoxy-benzaldehyde	C_18_H_18_O_7_	Petroleum ether	Silica gel column chromatography, RPC18, sephadex LH-20	MS, IR, ^1^H-NMR, ^13^C-NMR, ^1^H-^1^H COSY, HMQC, HMBC	Yu et al. (2014)
25	Hedyotol A	C_30_H_32_O_9_	Ethyl acetate	Silica gel/gel column chromatography, semi-preparative HPLC	MS, ^1^H-NMR, ^13^C-NMR	Hu (2016)
26	Picrasmalignan	C_30_H_30_O_9_	Ethyl acetate	Silica gel/gel column chromatography, semi-preparative HPLC	MS, ^1^H-NMR, ^13^C-NMR	Hu (2016)
27	Balanophonin	C_20_H_20_O_6_	Ethyl acetate	Silica gel/gel column chromatography, semi-preparative HPLC	MS, ^1^H-NMR, ^13^C-NMR	Hu (2016)
28	1-Propanone, 3-hydroxy-1-(4-hydrpxy-3-methoxyphenyl)-2-[4-(3-hydroxy-1-propen-1-yl)-2-methoxyphenoxy]	C_21_H_24_O_6_	Ethyl acetate	Silica gel/gel column chromatography, semi-preparative HPLC	MS, ^1^H-NMR, ^13^C-NMR	Hu (2016)
29	Erythro-guaiacylglycerol-b-coniferyl ether	C_20_H_24_O_7_	Ethyl acetate	Silica gel/gel column chromatography, semi-preparative HPLC	MS, ^1^H-NMR, ^13^C-NMR	Hu (2016)
30	Threo-guaiacylglycerol- b-coiferyl ether	C_20_H_24_O_7_	Ethyl acetate	Silica gel/gel column chromatography, semi-preparative HPLC	MS, ^1^H-NMR, ^13^C-NMR	Hu (2016)
31	(7R,8S)-Dehydrodiconiferyl alcohol γ′- methyl ether	C_21_H_24_O_6_	Ethyl acetate	Silica gel/gel column chromatography, semi-preparative HPLC	MS, ^1^H-NMR, ^13^C-NMR	Hu (2016)
32	3-Benzofuranmethanol, 2, 3-dihydro-2-(4-dydroxy-3-methoxypenyl)-7-methoxy-5-(3-methoxyl-1-propenyl)-,[2S-[2a,3b, 5(E)]]-(9CI)	C_21_H_24_O_6_	Ethyl acetate	Silica gel/gel column chromatography, semi-preparative HPLC	MS, ^1^H-NMR, ^13^C-NMR	Hu (2016)
33	Evofolin-B	C_17_H_18_O_6_	Ethyl acetate	Silica gel/gel column chromatography, semi-preparative HPLC	MS, ^1^H-NMR, ^13^C-NMR	Hu (2016)
34	1-Propanon, 3-hydroxy-1-(2-hydrpxy-5-methoxyphenyl)-2-(4-hydroxy-3-methoxyphenyl)-	C_20_H_24_O_7_	Ethyl acetate	Silica gel/gel column chromatography, semi-preparative HPLC	MS, ^1^H-NMR, ^13^C-NMR	Hu (2016)
35	Herpetatol A	C_19_H_18_O_5_	Ethyl acetate	Silica gel/gel column chromatography, semi-preparative HPLC	MS, ^1^H-NMR, ^13^C-NMR	Hu (2016)
36	Herpetatol B	C_19_H_16_O_5_	Ethyl acetate	Silica gel/gel column chromatography, semi-preparative HPLC	MS, ^1^H-NMR, ^13^C-NMR	Hu (2016)
37	Herpetatol C	C_20_H_22_O_7_	Ethyl acetate	Silica gel/gel column chromatography, semi-preparative HPLC	MS, ^1^H-NMR, ^13^C-NMR	Hu (2016)
38	Herpetatol D	C_31_H_32_O_9_	Ethyl acetate	Silica gel/gel column chromatography, semi-preparative HPLC	MS, ^1^H-NMR, ^13^C-NMR	Hu (2016)
39	Herpetatol E	C_30_H_30_O_9_	Ethyl acetate	Silica gel/gel column chromatography, semi-preparative HPLC	MS, ^1^H-NMR, ^13^C-NMR	Hu (2016)
40	Herpetatol F	C_29_H_28_O_8_	Ethyl acetate	Silica gel/gel column chromatography, semi-preparative HPLC	MS, ^1^H-NMR, ^13^C-NMR	Hu (2016)
41	Herpetatol G	C_29_H_28_O_8_	Ethyl acetate	Silica gel/gel column chromatography, semi-preparative HPLC	MS, ^1^H-NMR, ^13^C-NMR	Hu (2016)
42	(−)-pinoresinol monomethyl ether	C_21_H_24_O_6_	Ethyl acetate	Normal phase silica gel column chromatography, MPLC, semi-preparative HPLC	MS, IR,^1^H-NMR, ^13^C-NMR	Ma (2020)
43	epipinoresinol	C_20_H_22_O_6_	Ethyl acetate	Normal phase silica gel column chromatography, MPLC, semi-preparative HPLC	UV, MS, ^1^H-NMR, ^13^C-NMR	Ma (2020)
44	(+)-pinoresinol	C_20_H_22_O_6_	Ethyl acetate	Normal phase silica gel column chromatography, MPLC, semi-preparative HPLC	MS, IR, ^1^H-NMR, ^13^C-NMR	Ma (2020)
45	(+)-menbrine	C_21_H_24_O_5_	Ethyl acetate	Normal phase silica gel column chromatography, semi-preparative HPLC	MS, IR, ^1^H-NMR, ^13^C-NMR	Ma (2020)
46	cinncassins D	C_28_H_28_O_9_	Ethyl acetate	Normal phase silica gel column chromatography, semi-preparative HPLC	MS, UV, IR, ^1^H-NMR, ^13^C-NMR	Ma (2020)
47	(7R,7′R,7″R,8S,8′S,8″S)-4′,4″-dihydroxy-3,3′,3″,5-tetramethoxy-7,9':7′,9-diepoxy-4,8″-oxy-8,8′-sesqoineolignan-7″,9″-diol	C_31_H_36_O_11_	Ethyl acetate	Normal phase silica gel column chromatography, MPLC, sephadex LH-20, semi-preparative HPLC	MS, ^1^H-NMR, ^13^C-NMR	Ma (2020)
48	3-Benzofuranmethanol-2,3-dihydro-2-(4-hydroxy-3-methoxyphenyl)-4-:methoxy-6-[tetra-hydro-2-(3-hydroxy-4-methoxyphenyl)-3-methanol]-2-furanmethyl	C_31_H_36_O_8_	Ethyl acetate	Normal phase silica gel column chromatograpy	MS, ^1^H-NMR, ^13^C-NMR	Yuan et al. (2006b)
49	Ehletianol C	C_30_H_36_O_10_	Ethyl acetate	Normal phase silica gel column chromatography, semi-preparative HPLC	MS, UV, ^1^H-NMR, ^13^C-NMR	Ma (2020)
50	Herpetosiol G	C_20_H_22_O_5_	Ethyl acetate	Normal phase silica gel column chromatography, MPLC, semi-preparative HPLC	MS, ^1^H-NMR, ^13^C-NMR, HMBC	Ma (2020)
51	Herpetosiol H	C_23_H_30_O_8_	Ethyl acetate	Normal phase silica gel column chromatography, sephadex LH-20, semi-preparative HPLC	UV, IR, MS, ^1^H-NMR, ^13^C-NMR, HSQC, HMBC, COSY, NOESY	Ma (2020)
52	Herpetosiol I	C_30_H_34_O_10_	Ethyl acetate	Normal phase silica gel column chromatography, sephadex LH-20, semi-preparative HPLC	UV, IR, MS, ^1^H-NMR, ^13^C-NMR, HSQC, HMBC, COSY, NOESY	Ma (2020)
53	Herpetosiol J	C_23_H_24_O_9_	Ethyl acetate	Normal phase silica gel column chromatography, semi-preparative HPLC	UV, MS, ^1^H-NMR, ^13^C-NMR, HSQC, HMBC, COSY, NOESY	Ma (2020)
54	Herpetosiol K	C_30_H_30_O_9_	Ethyl acetate	Normal phase silica gel column chromatography, semi-preparative HPLC	UV, IR, MS, ^1^H-NMR, ^13^C-NMR, HSQC, HMBC, COSY, NOESY	Ma (2020)
55	Herpetosiol L	C_19_H_18_O_6_	Ethyl acetate	Normal phase silica gel column chromatography, MPLC, semi-preparative HPLC	UV, MS, ^1^H-NMR, ^13^C-NMR, HSQC, HMBC, COSY, NOESY	Ma (2020)
56	Herpetosiol M	C_20_H_20_O_7_	Ethyl acetate	Normal phase silica gel column chromatography, MPLC, semi-preparative HPLC	MS, ^1^H-NMR, ^13^C-NMR, HMBC	Ma (2020)
57	Herpetosiol N	C_32_H_38_O_11_	Ethyl acetate	Normal phase silica gel column chromatography, MPLC, sephadex LH-20, semi-preparative HPLC	MS, ^1^H-NMR, ^13^C-NMR, HSQC, HMBC, COSY, NOESY	Ma (2020)
58	Phyllanglaucin B	C_30_H_34_O_9_	Ethyl acetate	Normal phase silica gel column chromatography, recrystallization	MS, ^1^H-NMR, ^13^C-NMR	Huang et al. (2021)
59	Buddlenol E	C_32_H_38_O_10_	Ethyl acetate	Normal phase silica gel column chromatography, sephedax LH-20, semi-preparative HPLC	MS, ^1^H-NMR, ^13^C-NMR	Huang et al. (2021)
60	Spathulated	C_30_H_34_O_9_	Ethyl acetate	—	HPLC	Wei et al. (2020)
61	Threo-buddlenol E	C_31_H_36_O_11_	Ethyl acetate	—	HPLC	Wei et al. (2020)
62	Picrasmalignan A	C_29_H_28_O_9_	Ethyl acetate	—	HPLC	Wei et al. (2020)
63	9,3′-Dimethoxyhierochin A	C_21_H_24_O_6_	Ethyl acetate	—	HPLC	Wei et al. (2020)
64	Sesquilignan	C_30_H_34_O_9_	Ethyl acetate	—	HPLC	Yuan et al. (2019)
65	Herpedulin A	C_50_H_52_O_16_	Ethyl acetate	Silica gel column chromatography, preparative TLC	MS, ^1^H-NMR, ^13^C-NMR, HMBC	Meng et al. (2022)
66	Herpedulin B	C_30_H_34_O_10_	Ethyl acetate	Silica gel column chromatography, sephadex LH-20, semi-preparative HPLC	MS, ^1^H-NMR, ^13^C-NMR, HMBC	Meng et al. (2022)
67	Herpedulin C	C_31_H_36_O_11_	Ethyl acetate	Silica gel column chromatography, MPLC, sephadex LH-20, semi-preparative HPLC	MS, ^1^H-NMR, ^13^C-NMR, ^1^H-^1^H COSY, HSQC, HMBC, CD spectrum	Meng et al. (2022)
68	Herpedulin D	C_31_H_36_O_11_	Ethyl acetate	Silica gel column chromatography, MPLC, sephadex LH-20, semi-preparative HPLC	MS, ^1^H-NMR, ^13^C-NMR, ^1^H-^1^H COSY, HSQC, HMBC, CD spectrum	Meng et al. (2022)
69	Herpedulin E	C_30_H_30_O_9_	Ethyl acetate	Silica gel column chromatography, semi-preparative HPLC	MS, ^1^H-NMR, ^13^C-NMR, HMBC, CD spectrum	Meng et al. (2022)
70	Herpedulin F	C_32_H_38_O_11_	Ethyl acetate	Silica gel column chromatography, semi-preparative HPLC	MS, ^1^H-NMR, ^13^C-NMR, HSQC, COSY	Meng et al. (2022)
71	Herpedulin G	C_30_H_32_O_11_	Ethyl acetate	Silica gel column chromatography, MPLC, sephadex LH-20, semi-preparative HPLC	MS, ^1^H-NMR, ^13^C-NMR, HSQC, CD spectrum	Meng et al. (2022)
72	Herpedulin H	C_30_H_32_O_11_	Ethyl acetate	Silica gel column chromatography, MPLC, sephadex LH-20, semi-preparative HPLC	MS, ^1^H-NMR, ^13^C-NMR, CD spectrum	Meng et al. (2022)
73	Herpedulin I	C_30_H_32_O_11_	Ethyl acetate	Silica gel column chromatography, MPLC, sephadex LH-20, semi-preparative HPLC	MS, ^1^H-NMR, ^13^C-NMR, CD spectrum	Meng et al. (2022)
74	Herpedulin J	C_23_H_24_O_9_	Ethyl acetate	Silica gel column chromatography, semi-preparative HPLC	MS, ^1^H-NMR, ^13^C-NMR, HSQC, NOSY, HMBC	Meng et al. (2022)
75	Herpedulin K	C_30_H_26_O_9_	Ethyl acetate	Silica gel column chromatography, recrystalization	MS, ^1^H-NMR, ^13^C-NMR, HSQC, HMBC	Meng et al. (2022)
76	Herpedulin L	C_23_H_30_O_8_	Ethyl acetate	Silica gel column chromatography, sephadex LH-20, semi-preparative HPLC	MS, ^1^H-NMR, ^13^C-NMR, HMBC, NOESY, CD spectrum	Meng et al. (2022)
77	Herpedulin M	C_19_H_18_O_6_	Ethyl acetate	Silica gel column chromatography, semi-preparative HPLC	MS, ^1^H-NMR, ^13^C-NMR, HSQC, ^1^H-^1^HCOSY, HMBC	Meng et al. (2022)
78	Herpedulin N	C_20_H_20_O_7_	Ethyl acetate	Silica gel column chromatography, semi-preparative HPLC	MS, ^1^H-NMR, ^13^C-NMR	Meng et al. (2022)
79	Herpedulin O	C_20_H_22_O_5_	Ethyl acetate	Silica gel column chromatography, semi-preparative HPLC	MS, ^1^H-NMR, ^13^C-NMR, HMBC, CD spectrum	Meng et al. (2022)
80	Herpedulin P	C_19_H_20_O_5_	Ethyl acetate	Silica gel column chromatography, semi-preparative HPLC	MS, ^1^H-NMR, ^13^C-NMR CD spectrum	Meng et al. (2022)
Fatty acids						
81	Palmitic acid	C_16_H_32_O_2_	Petroleum ether	—	GC-MS	Liu et al. (2005a)
82	Oleic acid	C_18_H_34_O_2_	Petroleum ether	—	GC-MS	Liu et al. (2005a)
83	Stearic acid	C_18_H_34_O_2_	Petroleum ether	—	GC-MS	Liu et al. (2005a)
84	Linoleic acid	C_18_H_32_O_2_	Petroleum ether	—	GC-MS	Zhang et al. (2004)
85	Linolenic acid	C_18_H_30_O_2_	Petroleum ether	Normal phase silica gel column chromatography, semi- preparative HPLC	MS, ^1^H-NMR, ^13^C-NMR	Dong et al. (2019)
86	Trilinolein	C_57_H_98_O_6_	Petroleum ether	Normal phase silica gel column chromatography, preparative HPLC	MS, ^1^H-NMR, ^13^C-NMR	Dong et al. (2019)
87	9-Octadecenoic acid	C_18_H_34_O_2_	Petroleum ether	—	GC-MS	Liu et al. (2005b)
88	Octadecanoic acid	C_18_H_36_O_2_	Petroleum ether	—	GC-MS	Liu et al. (2005a)
89	cis-5-Dodecaenoic acid	C_12_H_22_O_2_	Petroleum ethe	Silica gel column chromatography	MS, ^1^H-NMR, ^13^C-NMR	Chen (2020)
90	Dodecanoic acid	C12H24O2	Ethyl acetate	Silica gel column chromatography	MS, ^1^H-NMR, ^13^C-NMR	Xu, (2012)
91	10-Eicossenoic acid	C20H38O2	Ethyl acetate	Silica gel column chromatography, sephadex LH-20	MS, ^1^H-NMR, ^13^C-NMR	Xu, (2012)
Terpenoids						
92	Neocucurbitacin D	C_31_H_44_O_8_	90% EtOH	Silica gel column chromatography, sephadex LH-20, RP-HLPC	MS, IR, ^1^H-NMR, ^13^C-NMR, HMBC, NOESY	Jiang et al. (2020)
93	Cucurbitacin E	C_32_H_44_O_8_	90% EtOH	Silica gel column chromatography, sephadex LH-20, semi-preparative HPLC	MS, ^1^H-NMR, ^13^C-NMR	Jiang et al. (2020)
94	Cucurbitacin D	C_30_H_44_O_7_	90% EtOH	Silica gel column chromatography, semi-preparative HPLC	MS, ^1^H-NMR, ^13^C-NMR	Jiang et al. (2020)
95	Cucurbitacin B	C_31_H_44_O_8_	90% EtOH	Silica gel column chromatography, sephadex LH-20, semi-preparative HPLC	MS, ^1^H-NMR, ^13^C-NMR	Jiang et al. (2020)
96	Cucurbitacin I	C_30_H_46_O_7_	90% EtOH	Silica gel column chromatography, semi-preparative HPLC	MS, ^1^H-NMR, ^13^C-NMR	Jiang et al. (2020)
97	23, 24-Dihydroisocucurbitacin B	C_32_H_48_O_8_	90% EtOH	Silica gel column chromatography, sephadex LH-20, RP-HLPC	MS, ^1^H-NMR, ^13^C-NMR	Jiang et al. (2020)
98	Cucurbitacin M	C_30_H_44_O_6_	Ethyl acetate	Normal phase silica gel column chromatography, MPLC, semi-preparative HPLC	MS, ^1^H-NMR, ^13^C-NMR, DEPT, HMBC, ^1^H-H COSY	Ma (2020)
99	Herpetosin A	C_22_H_30_O_6_	Ethyl acetate	Silica gel column chromatography	UV, IR, MS, ^13^C-NMR, ^1^H-NMR	Xu (2012)
100	Cucurbitacin L	C_30_H_44_O_7_	Ethyl acetate	Silica gel column chromatography, Sephadex LH-20	MS, ^1^H-NMR	Dai et al. (2017)
101	Oleanic acid	C_30_H_48_O_3_	Ethyl acetate	Silica gel column chromatography, Sephadex LH-20	MS, ^1^H-NMR	Dai et al. (2017)
Coumarins						
102	Herpetolide A	C_16_H_14_O_6_	Ethyl acetate	Silica gel column chromatography, recrystallization	MS, IR, ^1^H-NMR, ^13^C-NMR, HMBC, NOESY, DEPT, HMQC	Zhang et al. (2008)
103	Herpetolide B	C_16_H_12_O_6_	Ethyl acetate	Silica gel column chromatography, recrystallization	MS, IR, ^1^H-NMR, ^13^C-NMR, HMBC	Zhang et al. (2008)
104	Herpetosperin A	C_22_H_24_O_11_	Ethyl acetate	Silica gel column chromatography, ODS silica gel CC, semi-preparative HPLC	MS, IR, ^1^H-NMR, ^13^C-NMR, HMBC	Xu et al. (2015)
105	Herpetosperin B	C_22_H_24_O_11_	Ethyl acetate	Silica gel column chromatography, ODS silica gel CC, semi-preparative HPLC	MS, IR, ^1^H-NMR, ^13^C-NMR, HMBC	Xu et al. (2015)
106	Herpetospin C	C_23_H_26_O_10_	Ethyl acetate	Reverse phase silica gel column chromatography	UV, IR, MS, ^13^C-NMR, ^1^H-NMR	Xu (2012)
107	Herpetolide H	C_19_H_16_O_6_	Ethyl acetate	Normal phase silica gel column chromatography, recrystallization	MS, ^1^H-NMR, ^13^C-NMR	Huang et al. (2021)
108	Herpetospin D	C_22_H_22_O_11_	Ethyl acetate	Normal phase silica gel column chromatography, recrystallization	MS, ^1^H-NMR, ^13^C-NMR	Xu (2012)
Others						
109	Arginine	C_6_H_14_N_4_O_2_	HCl	—	HITACHI 835-50 High-speed amino acid analyzer, XDY-I atomic fluorescence spectrometer	Li et al. (2005)
110	Histidine	C_6_H_9_N_3_O_2_	HCl	—	HITACHI 835-50 High-speed amino acid analyzer, XDY-I atomic fluorescence spectrometer	Li et al. (2005)
111	Lysine	C_6_H_14_N_2_O_2_	HCl	—	HITACHI 835-50 High-speed amino acid analyzer, XDY-I atomic fluorescence spectrometer	Li et al. (2005)
112	Leucine	C_6_H_13_NO_2_	HCl	—	HITACHI 835-50 High-speed amino acid analyzer, XDY-I atomic fluorescence spectrometer	Li et al. (2005)
113	Isoleucine	C_6_H_13_NO_2_	HCl	—	HITACHI 835-50 High-speed amino acid analyzer, XDY-I atomic fluorescence spectrometer	Li et al. (2005)
114	Tryptophan	C_11_H_12_N_2_O_2_	HCl	—	HITACHI 835-50 High-speed amino acid analyzer, XDY-I atomic fluorescence spectrometer	Li et al. (2005)
115	Kaempferitrin	C_27_H_30_O_14_	Ethyl acetate	Reversed phase silica gel column chromatography, semi-preparative HPLC	UV, IR, ^1^H-NMR, ^13^C-NMR	Fan et al. (2016)
116	3′-Hydroxydaidzein	C_15_H_10_O_5_	Ethyl acetate	Silica gel column chromatography, semi-preparative HPLC	MS, ^1^H-NMR	Dai et al. (2017)
117	Stigmasterol	C_29_H_48_O	Ethyl acetate	Silica gel column chromatography, recrystalization	MS, ^1^H-NMR, ^13^C-NMR	Liu et al. (2010)
118	β-Stigmasterol	C_29_H_48_O	Ethyl acetate	Silica gel column chromatography, Sephadex LH-20	MS, ^1^H-NMR, ^13^C-NMR	Gong (2013)
119	Spinasterol glucoside	C_35_H_56_O_6_	Ethyl acetate	Silica gel column chromatography, recrystalization	UV, IR, MS, ^1^H-NMR, ^13^C-NMR	Liu et al. (2010)
120	Arbutin, 1-acetate	C_14_H_18_O_8_	Ethyl acetate	Reversed phase silica gel column chromatography	Uv, IR, MS, ^1^H-NMR, ^13^C-NMR	Hu (2016)
121	Herpetolide C	C_16_H_14_O_6_	Petroleum ether	Silica gel column chromatography, sephadex LH-20, smi-preparative HPLC	UV, IR, MMS, ^1^H-NMR, ^13^C-NMR, HMQC, HMBC	Fan et al. (2016)
122	Eicosanoic acid, 2-propenyl ester	C_23_H_44_O_2_	Ethyl acetate	Silica gel column chromatography, sephadex LH-20	MS, ^1^H-NMR	Dai et al. (2017)
123	3-Dodecen-1-yne	C_12_H_20_	petroleum ether	—	GC-MS	Liu et al. (2005)
124	2,6,10,14,18,22-Tetracosahexaen	C_24_H_38_	petroleum ether	—	GC-MS	Zhang et al. (2004)
125	Herpecaudin	C_17_H_22_O_4_	Ethyl acetate	RPHPLC, silica gel column chromatography, RP-18, sephadex LH-20	MS,1H-NMR,13C-NMR, HMBC, NOESY, CD spectrum, X-ray	Jiang et al. (2016)

### 4.1 Lignans

As collected in Figure 2 and summarized in Figure 3, lignans in *H. pedunculosum* seeds can be mainly divided into benzofurans, tetrahydrofurans and furofuran. In benzofuran lignan such as dehydrodiconiferyl alcohol (9) and herpetotriol (12), the benzene ring is linked to the side chain to form the furan oxygen ring (Figure 2; Table 1). In furfuran lignan, bimolecular phenylpropanin side chains are connected to form a *bis*-tetrahydrofuran ring, such as herpetetradione (4), herpetetrone (5) and herpetrione (6). Tetrahydrofurans lignans can be further divided into three types with 7-O-7' (**a**), 7-O-9' (**b**), and 9-O-9' (**c**) structures (Figure 2). The tetrahydrofuran of 7-O-9′ is predominant in *H. pedunculosum* seeds, represented by herpetriol (1) and herpetetrol (2). Beside above three main lignan types, *H. pedunculosum* seeds also contains dibenzylbutane (chemicals of 29 and 30) as shown in Figure 2.

### 4.2 Fatty acids

It has been found that *H. pedunculosum* seeds contain various fatty acids (81-91, Figure 4; Table 1), with comparatively greater concentrations of linoleic (84) and linolenic acid (85) (Zhao et al., 2009). Oleic (82), palmitic (81), and linoleic acids (84) are reported to be physiologically active in decreasing blood cholesterol levels and alleviating the formation of cholesterol in the vascular wall (Dobrzyńska and Przysławski, 2020). Therefore, it is essential to study the fatty acids in *H. pedunculosum* seeds.

### 4.3 Terpenoids

Ten terpenoids (92-101, Figure 5) were identified in *H. pedunculosum* seeds, and triterpenoid was the dominant type among them. Triterpenoids have the activities of anti-inflammatory, antibacterial, and antiviral properties (Xiao et al., 2018). For example, cucurbitacin B (95) was reported to show anti-inflammatory, antioxidant, and neuroprotective effects (Dai et al., 2023). These bioactive triterpenoids in *H. pedunculosum* seeds doubtlessly contribute to its favorable pharmacologic actions.

### 4.4 Coumarins

Coumarin is widely acknowledged to have extensive biological activities including anti-tumor, anti-oxidation, anti-inflammation, and anti-coagulation (Kirsch et al., 2016; Wu et al., 2020). And there are 7 coumarins (102-108 in Figure 5) found in *H. pedunculosum* seeds up to now. For example, Huang et al. (2021) found that herpetolide H (107) from *H. pedunculosum* seeds had the effects of anti-inflammatory *in vitro*.

### 4.5 Others

In addition to the aforementioned metabolites, *H. pedunculosum* seeds also contain amino acids (109–114), flavonoids (115, 116), sterols (117–119), glucosides (120), esters (121, 122), olefin (123–124), and ketones (125) as illustrated in Figure 6. It is reported that leucine (112) and isoleucine (113) can prevent the fat accumulation from in hepatocyte (Zhang et al., 2022). Kaempferitrin (115) has anti-inflammatory and anti-oxidation effects (Patel D. K., 2021). The biological activity of stigmasterol (117) is found to include anti-inflammatory, antioxidant, and anti-cancer properties (Bakrim et al., 2022). Therefore, the role of these metabolites in the application of *H. pedunculosum* seeds deserves further research.

## 5 Pharmacology

Diverse studies have demonstrated the hepatoprotective, antioxidant, and anti-cholestasis effects of *H. pedunculosum* seeds and aforementioned metabolites. Especially, the action mechanism on liver protection effect of *H. pedunculosum* seeds was systematically generalized. The specific hepatoprotective action and other pharmacological effects were summarized in Table 2 and Table 3, respectively.

**TABLE 2:** The hepatoprotective pharmacology of *H. pedunculosum* seeds.

Liver disease	Extract/Compound	Animal/cell and intervention	Indicators and results (control, model, treatment, positive control groups)	Refs.
Hepatic fibrosis	Ethyl acetate (EAEHPS)	Animal: Sprague-Dawley rats (male) Model: Induction of CCl_4_ (50%, 3 mL/kg) Treatment: EAEHPS (1 and 3 g/kg) for 6 weeks Positive control: Silymarin (0.1 g/kg) for 6 weeks	ALT↓, AST↓, TNF-a↓, IL-1β↓, IL-6↓, TGF-β1↓, NF-κB 65↓, IκBα↑, Smad3↑.(Compared with the model group) HA/μg^**.**^L^-1^: 49.35 ± 5.26, 75.37 ± 22.65, 61.27 ± 8.46 (L) 54.97 ± 8.63 (H), 39.94 ± 12.61; LN/μg^**.**^L^-1^: 73.55 ± 13.06, 131.74 ± 20.94, 110.38 ± 27.89 (L), 108.78 ± 6.61 (H), 112.87 ± 16.94; PCⅢ/μg^**.**^L^-1^: 75.57 ± 5.11, 117.65 ± 29.45, 98.38 ± 10.28 (L), 93.11 ± 10.19 (H), 88.60 ± 6.92; ColⅣ/μg L^-1^: 58.75 ± 23.14, 78.15 ± 14.70, 54.86 ± 16.03 (L), 46.31 ± 10.88 (H), 56.75 ± 15.14	Feng et al. (2018a)
	Chloroform	Animal: Sprague-Dawley rats (male) Model: Induction of CCl_4_ (50%, 3 mL/kg) Treatment: 1 and 3 g/kg) for 10 weeks	GPT↓, GOT↓, TBIL↓, CP↓, HA↓, LN↓, PCIII↓, ColIV↓, TBA↓, MDA↓, CAT↑, SOD↑, ALB↑.(Compared with the model group)	Li et al. (2019)
Hepatic fibrosis	Chloroform	Animal: KM mice Model: Induction of CCl_4_ (1%, 5 mL/kg) Treatment: 10 (L), 30 (M), 60 (H) g/kg for 1 week	ALT/U^**.**^L^-1^: 26.07 ± 3.23, 121.04 ± 9.8, 53.99 ± 3.21 (L), 37.25 ± 9.80 (M), 30.40 ± 2.44 (H),/); AST/U^**.**^L^-1^: 66.09 ± 8.99, 231.84 ± 18.32, 139.67 ± 13.98 (L), 126.63 ± 8.53 (M), 99.63 ± 36.89 (H),/; MDA/nmol^**.**^mg^-1^: 6.35 ± 1.49, 11.74 ± 1.07, 8.80 ± 1.87 (L) 7.77 ± 0.32 (M) 7.01 ± 0.48 (H),/; SOD/U^**.**^mg^-1^: 45.20 ± 6.00, 22.80 ± 4.3, 30.99 ± 2.80 (L), 41.06 ± 1.73 (M), 36.91 ± 7.89 (H),/; Caspase-3:/, 0.1674 ± 0.0061, 0.1555 ± 0.0010 (L), 0.1356 ± 0.0099 (M), 0.1096 ± 0.0083 (H),/	Jiang (2011)
Liver protection	Water	Animal: KM mice Model: Induction of CCl_4_ (1%, 5 mL/kg) Treatment: 10 (L), 30 (M), 60 (H) g/kg for 1 week	ALT/U^**.**^L^-1^: 26.07 ± 3.23, 121.04 ± 9.8, 72.16 ± 4.9 (L), 59.59 ± 9.81 (M), 54.92 ± 7.03 (H),/; AST/U^.^L^-1^: 66.09 ± 8.99, 231.84 ± 18.32, 185.29 ± 19.63 (L) 172.25 ± 12.61 (M), 160.15 ± 12.91 (H),/; MDA/nmol^**.**^mg^-1^: 6.35 ± 1.49, 11.74 ± 1.07, 8.80 ± 1.87 (L), 7.77 ± 0.32 (M), 7.01 ± 0.48 (H),/; SOD/U^**.**^mg^-1^: 45.20 ± 6.00, 22.80 ± 4.3, 30.99 ± 2.80 (L), 41.06 ± 1.73 (M), 36.91 ± 7.89 (H),/; Caspase-3:/, 0.1674 ± 0.0061, 0.1505 ± 0.0062 (L), 0.1366 ± 0.0012 (M), 0.1026 ± 0.0096 (H),/	
Chemical liver injury	Water	Animal: C57BL/6 male mice at 8–10 weeks of age Cell: BRL-3A and AML12 Model: Induction of APAP (300 mg/kg, 40 mM) Treatment: Water extract (0.3 mg/kg, 3 g/kg) in mice for 2 weeks; Water extract (100–400 μg/mL) in BRL-3A for 24 h. Water extract (100–400 μg/mL) in AML12 for 8 h	ALT↓, AST↓, ROS↓, TNF-α↓, 1L-1β↓, HO-1↓, NQO1↓, Cell viability↑, GSH↑	Li J. et al. (2023)
Drug-induced liver injury	Ethanol	Animal: C57BL/6 (male); Cell: BRL-3A Model: Induction of APAP (Cell: 40 mM, 8 h; Mice: 200 mg/kg); Treatment: ethanol extract (6.25, 12.5, 25 μg/mL) in BRL-3A; ethanol extract (0.3, 1, 3 g/kg) in mice for 15 days	ALT↓, AST↓, ROS↓, MDA↓, Bax↓, Caspase3↓, Cleaved Caspase3↓, HO-1↑, NQO1↑, Cell viability↑, GSH↑	Liao (2023)
Liver protection	Petroleum ether	Animal: Sprague-Dawley rats (male) Model: Induction of ANIT (60 mg/kg) Treatment: Petroleum ether extract of 350 (L), 700 (M), 1400 mg/kg (H) for 5 days Positive control: Ursodeoxycholic acid (UDCA) (100 mg/kg) for 5 days	ALT↓, AST↓, ALP↓, γ-GTP↓, TBIL↓, DBIL↓, TBA↓, degree of tissue damage↓.(Compared with the model group) MDA/nmol^**.**^mg^-1^: 1.24 ± 0.04, 4.02 ± 0.06, 3.91 ± 0.49 (L), 2.61 ± 0.32 (M), 1.84 ± 0.09 (H), 2.65 ± 0.28); MPO/U^**.**^mg^-1^: 3.70 ± 0.42, 24.10 ± 4.26, 23.44 ± 3.01 (L), 19.79 ± 1.74 (M), 12.13 ± 0.64 (H), 15.62 ± 0.75; NO/μmol^**.**^L^-1^: 5.007 ± 2.678, 4.006 ± 0.732 (L), 3.523 ± 0.223 (M), 3.351 ± 0.194 (H), 2.678 ± 0.375; SOD/U^**.**^mg^-1^: 166.81 ± 10.80, (56.07 ± 4.62, 57.11 ± 4.19 (L), 62.56 ± 4.44 (M), 84.52 ± 7.02 (H), 109.02 ± 12.21; GST/nmol^**.**^min^-1**.**^mg^-1^: 56.15 ± 6.39, 37.40 ± 2.85, 38.66 ± 3.92 (L), 43.75 ± 2.59 (M), 47.60 ± 1.66 (H), 47.93 ± 3.27; NO/: 1.884 ± 0.122, 5.007 ± 2.678, 4.006 ± 0.732 (L) 3.523 ± 0.223 (M), 3.351 ± 0.194(H), 2.678 ± 0.375	Cao et al. (2017)
Chemical liver injury	Total lignans	Animal: ICR mice (male) Model: Induction of CCl_4_ (0.1%, 20 mL/kg) Treatment: Total lignans (0.375, 0.75, 1.5, 3 g/kg) for 7 days Positive control: Compound glycyrrhizin tablets of 113 mg/kg (P1) and biphenyl diester of 200 mg/kg (P2) for 7 days	ALT/U^**.**^L^-1^: 50.68 ± 3.66, 259.70 ± 3.58, 231.81 ± 16.73 (0.375 g/kg) 210.71 ± 9.08 (0.75 g/kg) 218.25 ± 6.17 (1.5 g/kg) 202.86 ± 11.80 (3 g/kg), 194.85 ± 17.46 (P1) 220.29 ± 7.77 (P2); AST/U^.^L^-1^: 97.83 ± 8.04, 274.50 ± 7.35, 240.38 ± 12.23 (0.375 g/kg) 233.17 ± 17.42 (0.75 g/kg) 226.55 ± 16.93 (1.5 g/kg) 213.31 ± 27.07 (3 g/kg), 209.38 ± 11.61 (P1) 232.90 ± 11.61 (P2); ALP/U^.^L^-1^: 117.88 ± 12.99, 195.67 ± 16.08, 143.28 ± 12.46 (0.375 g/kg) 138.61 ± 10.53 (0.75 g/kg) 134.61 ± 12.73 (1.5 g/kg) 124.14 ± 14.72 (3 g/kg), 158.29 ± 9.55 (P1) 131.74 ± 21.67 (P2); MDA/nmol^**.**^mgprot^-1^: 12.54 ± 1.59, 35.32 ± 2.54, 23.64 ± 2.82 (0.375 g/kg) 20.72 ± 1.49 (0.75 g/kg) 19.73 ± 1.28 (1.5 g/kg) 16.03 ± 2.76 (3 g/kg), 17.43 ± 2.44 (P1) 20.67 ± 1.98 (P2); SOD/U^**.**^mgprot^-1^: 76.84 ± 3.59, 43.39 ± 1.72, 52.75 ± 2.58 (0.375 g/kg) 54.58 ± 3.24 (0.75 g/kg) 55.02 ± 1.20 (1.5 g/kg) 59.99 ± 2.35 (3 g/kg), 50.79 ± 1.93 (P1) 49.75 ± 1.93 (P2); GSH-Px/U^**.**^mgprot^-1^: 996.76 ± 81.60, 534.00 ± 50.58, 873.88 ± 96.38 (0.375 g/kg) 896.26 ± 151.70 (0.75 g/kg) 924.47 ± 125.97 (1.5 g/kg) 975.95 ± 152.21 (3 g/kg), 751.57 ± 46.27 (P1) 796.84 ± 83.47 (P2)	Zhao et al. (2015)
Hepatic fibrosis	Total lignans	Animal: Sprague-Dawley rats (male) Model: Induction of CCl_4_ (40%, 25 mg/kg) Treatment: Total lignans of 100 (L), 200 (M), 400 mg/kg (H) for 8 weeks	ALT/U^**.**^L^-1^: 82.25 ± 5.47, 200.00 ± 22.60, 139.86 ± 21.05 (L) 106.63 ± 16.60 (M) 92.75 ± 18.42 (H), 87.75 ± 114.47; AST/U^**.**^L^-1^: 169.25 ± 13.96, 217.57 ± 33.76, 225.86 ± 9.86 (L) 202.38 ± 38.03 (M) 178.00 ± 31.96 (H), 185.50 ± 30.87; ALP/U^**.**^L^-1^: 158.00 ± 4.04, 201.29 ± 25.45, 151.14 ± 226.17 (L) 171.25 ± 31.32 (M) 145.50 ± 18.53 (H), 136.50 ± 28.4; TGF-β1/ng^**.**^L^-1^: 173.37 ± 2.94, 225.15 ± 17.99, 210.64 ± 11.67 (L) 196.79 ± 15.77 (M) 188.32 ± 16.64 (H), 193.11 ± 13.22; HA/ng^**.**^L^-1^: 248.21 ± 9.99, 313.55 ± 16.29, 291.63 ± 11.37 (L) 273.21 ± 19.14 (M) 272.20 ± 21.30 (H), 271.04 ± 10.42; HYP/μg^**.**^L^-1^: 672.15 ± 10.85, 810.04 ± 25.60, 791.46 ± 21.34 (L) 742.96 ± 27.21 (M) 728.60 ± 40.68 (H), 725.27 ± 19.86; SOD/μg^**.**^L^-1^: 10.88 ± 0.28, 9.04 ± 0.46, 9.40 ± 0.46 (L) 10.02 ± 0.44 (M) 10.23 ± 0.67 (H), 10.23 ± 0.39	Liu et al. (2017a)
Acute alcoholic liver injury	Total lignans	Animal: KM mice (male) Model: 56° Beijing Red Star Erguotou wine Treatment: Total lignans of 15 (L), 25 (M), 35 mg/kg (H) for 30 days Positive control: Polyene phosphatidylcholine (135 mg/kg) for 30 days	AST/U^**.**^L^-1^: 143.7 ± 12.0, 258.7 ± 28.3, 230.0 ± 23.3 (L) 200.2 ± 25.5 (M) 222.2 ± 28.2 (H), 185.8 ± 39.6; ALT/U^**.**^L^-1^: 56.5 ± 6.5, 155.0 ± 27.8, 1123.3 ± 26.1 (L) 92.8 ± 14.7 (M) 98.5 ± 15.3 (H), 99.8 ± 9.6; MDA/[nmol^.^(mg^**.**^pro)^−1^]: 1.07 ± 0.14, 1.99 ± 0.87, 1.69 ± 1.26 (L) 1.14 ± 0.27 (M) 1.21 ± 0.28 (H), 1.22 ± 0.15; XOD/[U^.^(mg pro)^−1^]: 13.7 ± 1.3, 5.3 ± 3.1, 6.5 ± 1.2 (L) 8.6 ± 1.7 (M) 7.4 ± 1.0 (H), 9.9 ± 22.9; Na^+^-K^+^-ATP/[μmolPi^**.**^(mg^**.**^pro^**.**^h)^−1^]: 0.98 ± 0.14, 0.38 ± 0.06, 0.63 ± 0.18 (L) 0.76 ± 0.08 (M) 0.82 ± 0.10 (H), 0.75 ± 0.07; SOD/[U^.^(mg^**.**^pro)^−1^]: 65.8 ± 5.1, 61.2 ± 2.8 (L) 62.7 ± 5.7 (M) 60.0 ± 4.3 (H), 59.6 ± 2.8; GSH-Px/[U^.^(mg^**.**^pro)^−1^]: 21.1 ± 7.9, 9.5 ± 2.5, 14.3 ± 1.2 (L) 12.2 ± 1.7 (M) 13.4 ± 0.7 (H), 13.7 ± 1.0	Huang et al. (2017)
Chronic alcoholic liver injury		Animal: Wistar rats (male) Model: 56° liquor (8 mL/kg-15 mL/kg) for 8 weeks Treatment: Total lignans of 100 (L), 200 (M), 400 mg/kg (H) for 8 weeks Positive control: Yishanfu (95 mg/kg) for 8 weeks	AST/U^**.**^L^-1^: 24.42 ± 2.79, 58.21 ± 14.83, 41.21 ± 7.69 (L) 29.92 ± 2.99 (M) 25.69 ± 10.74 (H), 36.05 ± 15.47; ALT/U^**.**^L^-1^: 11.34 ± 0.69, 51.53 ± 2.18, 34.83 ± 4.77 (L) 27.45 ± 1.82 (M) 331.99 ± 2.30 (H), 331.68 ± 5.09; MDA/[nmol^.^(mg^**.**^prot)^−1^]: 1.15 ± 0.33, 3.35 ± 1.15, 1.57 ± 0.19 (L) 1.09 ± 0.29 (M) 1.17 ± 0.29 (H), 1.32 ± 0.31; ADH/[nmol/(min^**.**^mg pro)]: 3.83 ± 0.82, 12.38 ± 3.60, 7.75 ± 2.89 (L) 5.99 ± 1.77 (M) 6.91 ± 1.42 (H), 8.39 ± 44.43; TG/mmol^**.**^L^-1^: 7.63 ± 0.73, 10.62 ± 0.74, 9.55 ± 0.99 (L) 8.51 ± 0.67 (M) 8.75 ± 0.63 (H), 8.01 ± 1.67; SOD/[U^.^(mg^**.**^prot)^−1^]: 423.81 ± 75.64, 193.52 ± 40.85, 317.09 ± 52.41 (L) 233.66 ± 64.95 (M) 296.12 ± 34.64 (H), 196.23 ± 80.47; GSH/[mg^**.**^(g^**.**^prot)^−1^]: 4.47 ± 1.81, 1.47 ± 0.47, 2.77 ± 0.39 (L) 3.60 ± 0.33 (M) 2.93 ± 0.53 (H), 2.11 ± 1.04; GSH-Px/[U^**.**^ **(**mg^**.**^prot)^−1^]: 40.2 ± 4.45, 34.1 ± 3.85, 39.1 ± 4.85 (L) 39.5 ± 3.25 (M) 35 ± 2.71 (H), 41.5 ± 4.23; CAT/U^**.**^mL^-1^: 10.03 ± 1.13, 7.09 ± 1.26, 9.08 ± 0.51 (L) 9.17 ± 1.18 (M) 8.31 ± 0.95 (H), 9.36 ± 0.93; ALDH2/[nmol/(min^**.**^mg pro)]: 9.62 ± 1.96, 3.40 ± 1.33, 4.96 ± 1.59 (L) 5.78 ± 3.53 (M) 3.07 ± 1.37 (H), 5.83 ± 3.78	Huang et al. (2018)
Cholestatic liver injury		Animal: KM mice (male) Model: Induction of ANIT (0.4%, 80 mg/kg) Treatment: Total lignans (0.05, 0.1, 0.2, 0.4 g/kg) for 7 days Positive control: Bifendate Pills group (0.15 g/kg) for 7 days	AST/U^**.**^L^-1^: 36.81 ± 11.13, 197.99 ± 11.67, 173 ± 21.48 (0.05 g/kg) 127.02 ± 11.07 (0.1 g/kg) 120.56 ± 16.87 (0.2 g/kg) 107.67 ± 44.34 (0.4 g/kg), 156.83 ± 16.49; ALT/U^**.**^L^-1^: 26.87 ± 14.69, 470.15 ± 18.68, 275.82 ± 17.69 (0.05 g/kg) 223.29 ± 42.17 (0.1 g/kg) 206.47 ± 25.35 (0.2 g/kg) 384.08 ± 26.11 (0.4 g/kg), 220.50 ± 46.87; ALP/U^**.**^L^-1^: 3.4 ± 0.6, 18.27 ± 2.53, 13.40 ± 1.87 (0.05 g/kg) 11.89 ± 3.12 (0.1 g/kg) 9.98 ± 2.04 (0.2 g/kg) 11.91 ± 1.36 (0.4 g/kg), 8.26 ± 2.23; TBA/μmol^**.**^L^-1^: 3.63 ± 0.35, 78.10 ± 8.38, 48.13 ± 8.98 (0.05 g/kg) 44.13 ± 13.28 (0.1 g/kg) 31.83 ± 5.84 (0.2 g/kg) 50.57 ± 17.10 (0.4 g/kg), 26.94 ± 110.15; TBIL/μmol^**.**^L^-1^: 1.62 ± 0.66, 191.57 ± 34.47, 106.56 ± 22.48 (0.05 g/kg) 41.65 ± 17.54 (0.1 g/kg) 229.89 ± 17.11 (0.2 g/kg) 96.07 ± 18.03 (0.4 g/kg), 41.96 ± 24.65; DBIL/μmol^**.**^L^-1^: 0.87 ± 0.19, 124.94 ± 18.72, 27.23 ± 9.13 (0.05 g/kg) 16.76 ± 10.48 (0.1 g/kg) 10.91 ± 6.21 (0.2 g/kg) 48.08 ± 21.09 (0.4 g/kg), 8.98 ± 3.92; SOD/U^**.**^mg^-1^: 527.97 ± 18.82, 243.02 ± 31.43, 297.27 ± 24.09 (0.05 g/kg) 2,295.93 ± 20.08 (0.1 g/kg) 322.70 ± 20.08 (0.2 g/kg) 312.37 ± 15.70 (0.4 g/kg), 368.28 ± 15.36); MDA/nmol^**.**^mg^-1^: 2.41 ± 0.92, 12.66 ± 1.61, 6.91 ± 0.95 (0.05 g/kg) 8.02 ± 2.18 (0.1 g/kg) 5.69 ± 1.27 (0.2 g/kg) 8.21 ± 2.56 (0.4 g/kg), 5.93 ± 2.28; CAT/U^**.**^mg^-1^: 22.96 ± 1.17, 8.87 ± 1.26, 12.56 ± 1.39 (0.05 g/kg) 17.97 ± 5.30 (0.1 g/kg) 13.53 ± 4.83 (0.2 g/kg) 18.77 ± 3.78 (0.4 g/kg), 19.41 ± 3.14; GSH-Px/mg^.^g^-1^: 132.54 ± 24.50, 21.51 ± 8.74, 54.45 ± 14.00 (0.05 g/kg) 70.80 ± 9.17 (0.1 g/kg) 83.14 ± 24.01 (0.2 g/kg) 77.28 ± 10.77 (0.4 g/kg), 89.81 ± 30.43; TNF-α/ng^**.**^L^-1^: 43.63 ± 2.07, 65.14 ± 7.40, 52.38 ± 3.34 (0.05 g/kg) 48.20 ± 1.91 (0.1 g/kg) 45.81 ± 2.09 (0.2 g/kg) 46.75 ± 3.10 (0.4 g/kg), 44.22 ± 2.5; MCP-1/ng^**.**^L^-1^: 31.11 ± 2.34, 226.06 ± 43.42, 155.01 ± 30.14 (0.05 g/kg) 117.14 ± 24.86 (0.1 g/kg) 110.79 ± 19.70 (0.2 g/kg) 154.40 ± 36.39 (0.4 g/kg), 129.28 ± 32.20	Li et al. N. J. (2023)
acute alcoholic liver injury	Total sterols	Animal: ICR mice (male) Model: Induction of CCl_4_ (0.3%, 10 mL/kg) Treatment: Total sterols extract(10, 20, 50 mg/kg) for 7 days Positive control: Silymarin (50 mg/kg) for 7 days	AST↓, ALT↓, IL-1β↓, IL-6↓, COX-2↓, IL-10↑	Liu et al. (2022)
Immunological liver injury	Fatty acid	Animal: Swiss mice Model: 2.5 mg BCG was given by tail injection Treatment: Fatty acid extract of 7 (L), 10 (M), 14.5 mL/kg (H) for 12 days Positive control: Bifendate (200 mg/kg) for 12 days	MDA/nmol^**.**^mg^-1^: 16.26 ± 4.29, 20.56 ± 3.61, 16.51 ± 2.89 (L) 19.36 ± 3.01 (M) 19.29 ± 1.99 (H), 17.73 ± 1.01; ALT/U^**.**^L^-1^: 7.67 ± 1.27, 90.71 ± 16.62, 23.32 ± 8.30 (L) 45.83 ± 16.92 (M) 29.16 ± 16.90 (H), 18.84 ± 8.73; AST/U^**.**^L^-1^: 23.48 ± 4.39, 92.39 ± 10.81, 47.61 ± 5.37 (L) 51.54 ± 13.11 (M) 44.72 ± 15.61 (H), 54.44 ± 17.37; NO/μmol^**.**^L^-1^: 3.33 ± 1.69, 21.26 ± 8.20, 8.45 ± 2.13 (L) 14.21 ± 9.43 (M) 10.77 ± 3.70 (H), 7.38 ± 4.66; SOD/U^.^mg^-1^: 184.40 ± 17.25, 105.00 ± 22.71, 219.95 ± 16.13 (L) 196.19 ± 23.09 (M) 228.28 ± 27.69 (H), 127.89 ± 12.69	Chen et al. (2014)
Liver protection		Animal: Sprague-Dawley rats (male) Model: Induction of CCl_4_ (3 mL/kg) Treatment: Fatty acid extract of 1(L), 2 (M), 4 g/kg (H) for 5 days Positive control: Bifendate (200 mg/kg) for 5 days	TG/nmol^**.**^L^-1^: 0.67 ± 0.21, 2.18 ± 0.53, 1.10 ± 0.38 (L) 1.03 ± 0.40 (M) 0.82 ± 0.1 (H), 1.44 ± 0.34; HDL/nmol^**.**^L^-1^: 0.99 ± 0.19, 1.30 ± 0.11, 0.43 ± 0.32 (L) 0.50 ± 0.13 M) 0.59 ± 0.12 (H), 0.57 ± 0.11; LDL/nmol^**.**^L^-1^:1.03 ± 0.24, 1.65 ± 0.10, 1.11 ± 0.37 (L) 1.08 ± 0.33 (M) 1.14 ± 0.20 (H), 1.06 ± 0.19; MDA/[nmol^.^(mg prot^-1^)]: 1.30 ± 0.11, 0.43 ± 0.32 (L) 0.50 ± 0.13 (M) 0.59 ± 0.12 (H), 0.57 ± 0.11; SOD/U^**.**^L^-1^: 57.69 ± 15.08, 42.86 ± 10.76, 74.28 ± 17.91 (L) 97.30 ± 12.51 (M) 102.69 ± 29.39 (H), 100.57 ± 21.66; TBIL/Umol^**.**^L^-1^: 1.15 ± 0.98, 11.89 ± 3.87, 6.54 ± 1.58 (L) 5.67 ± 2.07 (M) 4.75 ± 1.09 (H), 9.7 ± 2.6; AST/U^**.**^L^-1^: 229.00 ± 35.03, 1084.86 ± 289.13, 1181.38 ± 178.33 (L) 1039.43 ± 244.18 (M) 310.10 ± 33.99 (H), 1394.20 ± 278.11; ALT/U^**.**^L^-1^: 39.60 ± 5.41, 1263.43 ± 361.30, 1285.38 ± 322.05 (L) 1109.14 ± 365.50 (M) 297.40 ± 76.87 (H), 1394.20 ± 278.11; ALP/U^**.**^L^-1^: 136.7 ± 23.3, 281.6 ± 36.30, 220.8 ± 34.3 (L) 185.0 ± 21.3 (M) 141.3 ± 27.8 (H), 191.4 ± 29.4	Li et al. (2014)
Immunological liver injury	Polysaccharide	Animal: KM mice (male) Model: ConA (30 mg/kg) injection into the tail vein Treatment: Polysaccharide of 0.71 (L), 0.99 (M), 1.44 g/kg (H) for 8 days Positive control: Bifendate (0.2 g/kg) for 8 days	ALT/U^**.**^L^-1^: 9.0 ± 0.8, 143.6 ± 7.0, 130.2 ± 6.2(L) 115.9 ± 9.4 (M) 45.8 ± 4.7 (H), 42.6 ± 6.0; AST/U^**.**^L^-1^: 24.6 ± 2.4, 172.3 ± 9.4, 146.2 ± 15.4 (L) 124.2 ± 8.0 (M) 55.9 ± 4.4 (H), 172.3 ± 9.4; LDH/U^**.**^L^-1:^ 1952.7 ± 133.7, 4606.6 ± 191.6, 3,948.3 ± 232.1 (L) 3,814.3 ± 227.8 (M) 3,187.9 ± 192.9 (H), 2,742.9 ± 179.3; NO/μmol^**.**^L^-1^: 2.3 ± 0.2, 6.7 ± 0.5, 4.5 ± 0.3 (L) 4.1 ± 0.5 (M) 3.6 ± 0.4 (H) 3.4 ± 0.2; IL-6/pg^**.**^mL^-1^: 30.4 ± 1.1, 74.5 ± 2.1, 56.5 ± 3.7 (L) 50.1 ± 2.8 (M) 45.7 ± 2.9 (H), 51.4 ± 3.2; MDA/[(nmol/mg^**.**^prot)]: 6.5 ± 0.3, 9.3 ± 0.5, 11.2 ± 0.7 (L) 9.0 ± 0.4 (M) 6.4 ± 0.7 (H), 7.5 ± 0.3; SOD/[(U/mg^**.**^prot)]: 187.6 ± 4.4 59.7 ± 4.3, 74.6 ± 3.1 (L) 99.9 ± 7.0 (M) 126 ± 10.7 (H), 90.9 ± 10.0; Degree of tissue damage↓	Li et al. (2015a)
Acute alcoholic liver injury	Herpetfluorenone	Animal: C57BL/6 mice; Cell: BMSCs Model: Induction of CCl_4_ Treatment: 100 μM of Herpetfluorenone	AST↓, ALT↓, ALP↓, TBA↓, MDA↓, ALB↑, SOD↑, GSH↑	Yang et al. (2023)
Acute alcoholic liver injury	Herpetin	Animal: C57BL/6 mice (male)Cell: BMSCs; Model: Induction of CCl_4_ Treatment: 10 μM of Herpetin	AST↓, ALT↓, AKP↓, ALB↑	Ding et al. (2023)
Immunological liver injury	Herpetin	Animal: ICR mice (male); Model: ConA (20 mg/kg) injection into the tail vein; Treatment: 10 (L), 20 mg/kg (H) of herpetin for 7 days Positive control: Qingkailing injection (20 mg/kg) for 5 days	iNOS: 0.215 ± 0.004, 0.290 ± 0.013, 0.275 ± 0.012 (L) 0.239 ± 0.009 (H), 0.237 ± 0.008; TNF-α: 0.130 ± 0.006, 0.166 ± 0.008, 0.145 ± 0.004 (L) 0.139 ± 0.005 (H), 0.141 ± 0.005; NF-κB: 0.129 ± 0.006, 0.150 ± 0.004, 0.153 ± 0.006 (L) 0.130 ± 0.002 (H), 0.141 ± 0.003; IFN-γ: 0.131 ± 0.006, 0.149 ± 0.006, 0.134 ± 0.003 (L) 0.132 ± 0.003 (H), 0.132 ± 0.005; IL-4: 0.104 ± 0.002, 0.129 ± 0.004, 0.121 ± 0.004 (L) 0.118 ± 0.002 (H) 0.117 ± 0.003; SOCS1: 0.120 ± 0.007, 0.081 ± 0.005, 0.087 ± 0.007 (L) 0.091 ± 0.008 (H), 0.105 ± 0.011	Wang et al. (2016)
Immunological liver injury	Herpetin	Animal: ICR mice (male) Model: Induction of BCG (2.5 mg) +LPS (7.5 μg) Treatment: 10 (L), 20 mg/kg (H) of herpetin for 12 days Positive control: Qingkailing injection (20 mg/kg) for 12 days	AST/U^**.**^L^-1^: 96.01 ± 9.40, 197.55 ± 8.1, 184.33 ± 15.86 (L) 161.59 ± 18.20 (H), 123.55 ± 11.07; ALT/U^**.**^L^-1^: 42.24 ± 1.52, 101.61 ± 5.05, 92.69 ± 2.75 (L), 68.35 ± 0.94 (M), 58.32 ± 2.44; LDH/U^**.**^L^-1^: 239.11 ± 20.05, 546.87 ± 18.16, 481.84 ± 9.04 (L) 393.70 ± 32.96 (H), 340.78 ± 16.13; MDA/nmol^**.**^mgprot^-1^: 15.98 ± 1.39, 38.41 ± 1.59, 35.61 ± 1.87 (L) 29.6 ± 1.52 (H), 24.38 ± 2.03; SOD/U^**.**^(mg^**.**^prot^-1^:97.47 ± 9.15, 68.08 ± 12.80, 75.75 ± 9.09 (L) 85.04 ± 8.75 (H), 88.64 ± 11.92; GSH-Px/U^**.**^mg^**.**^prot^-1^:545.37 ± 54.86, 292.78 ± 57.38, 380.12 ± 33.94 (L) 414.53 ± 48.03 (H), 463.56 ± 32.30	Liu (2017b)

**TABLE 3:** The other pharmacology effects of *Herpetospermum pedunculosum* seeds.

Effects	Extract/Compound	Animal/Cell and intervention	Indicators and results (control, model, treatment, positive control groups)	Refs.
Antioxidation	Chloroform	Animal: SD rats Model: Induction of CCl_4_ (50%, 0.6 mg/kg) Treatment: chloroform extract of 200 (L), 400 mg/kg (H) for 7 days. Water extract of 200 (L), 400 mg/kg (H) for 7 days Positive control: VitE (400 mg/kg) for 7 days	MDA/nmol^**.**^mg^-1^ protein: 8.65 ± 2.89, 16.92 ± 4.75, 7.22 ± 1.94 (L) 7.34 ± 0.97 (H), 5.16 ± 0.64; SOD/unit^**.**^mg^-1^ protein: 316.68 ± 19.05, 237.62 ± 17.81, 292.83 ± 41.64 (L) 289.52 ± 40.07 (H), 289.00 ± 25.29; GSH-px/unit^**.**^mg^-1^ protein: 146.36 ± 24.67, 101.82 ± 24.17, 118.51 ± 18.36 (L) 121.68 ± 223.16 (H), 142.56 ± 16.61	Fang et al. (2008)
	Water		MDA/nmol^**.**^mg^-1^ protein: 8.65 ± 2.89, 6.89 ± 1.26 (L) 6.11 ± 0.48 (H), 5.16 ± 0.64 SOD/unit^**.**^mg^-1^ protein: 316.68 ± 19.05, 272.29 ± 25.97 (L) 308.15 ± 13.34 (H), 289.00 ± 25.29; GSH-px/unit^**.**^mg^-1^ protein: 146.36 ± 24.67, 142.48 ± 10.83 (L) 148.25 ± 12.35 (H), 142.56 ± 16.61	
Anti-fatigue	Chloroform	Animal: KM mice (the mice that could learn to swim, male) Treatment: chloroform extract of 80 (L), 160 (M), 320 mg/kg (H); Ethyl acetate extract of 80 (L), 160 (M), 320 mg/kg (H); n-Butanol extract of 80 (L), 160 (M) 320 mg/kg (H) for 30 days; herpetrione of 15 (L), 30 (M), 60 mg/kg(H) for 30 days	Swimming time↑, survival time↑; HG/mg^**.**^g^-1^:9.99 ± 1.58,/, 10.40 ± 1.47 (L) 10.53 ± 1.56 (M) 10.58 ± 1.97 (H),/; LDH/U^**.**^L^-1^: 874.50 ± 64.22,/, 900.56 ± 143.87 (L) 942.11 ± 127.10 (M) 961.84 ± 70.95 (H),/; SOD/U^**.**^mL^-1^: 69.52 ± 9.79,/, 119.84 ± 16.13 (L) 118.50 ± 9.52 (M) 121.28 ± 8.44 (H),/; GSH-Px/U^**.**^L^-1^: 109.56/, 119.84 ± 16.13 (L) 118.50 ± 9.52 (M) 121.28 ± 8.44 (H),/; BLA/ng^.^100mL^-1^: 24.49 ± 1.99,/, 23.91 ± 2.85 (L) 23.19 ± 1.84 (M) 23.37 ± 1.67 (H),/; MDA/nmol^**.**^L^-1^:14.04 ± 2.07,/, 13.92 ± 1.58 (L) 13.56 ± 1.91 (M) 13.43 ± 1.42 (H),/	Jin et al. (2016)
	Ethyl acetate		Swimming time↑, survival time↑; HG/mg^**.**^g^-1^: 9.99 ± 1.58,/, 10.55 ± 1.60 (L) 10.90 ± 1.58 (M) 11.56 ± 1.28 (H),/; LDH/U^**.**^L^-1^: 874.50 ± 64.22,/, 916.63 ± 137.80 (L) 996.50 ± 112.53 (M) 1073.66 ± 140.79 (H),/; SOD/U^**.**^mL^-1^ : 69.52 ± 9.79,/, 118.69 ± 9.38 (L) 120.54 ± 11.04 (M) 123.78 ± 8.18 (H),/; GSH-Px/U^**.**^L^-1^: 109.56 ± 9.58,/, 118.69 ± 9.38 (L) 120.54 ± 11.04 (M) 123.78 ± 8.18 (H) 1,/; BLA/ng^.^100mL^-1^: 24.49 ± 1.99,/, 23.09 ± 1.70 (L) 22.16 ± 2.16 (M) 21.94 ± 2.24 (H),/; MDA/nmol^**.**^L^-1^: 14.04 ± 2.07,/, 13.26 ± 1.47 (L) 12.261 ± 1.13 (M) 11.77 ± 1.44 (H),/	
	n-Butanol		HG/mg^**.**^g^-1^: 9.99 ± 1.58,/, 10.04 ± 1.44 (L) 10.40 ± 1.79 (M) 11.45 ± 1.14 (H),/; LDH/U^**.**^L^-1^: 874.50 ± 64.22,/, 914.42 ± 153.03 (L) 926.89 ± 111.32 (M) 1028.14 ± 104.08 (H),/; SOD/U^**.**^mL^-1^: 69.52 ± 9.79,/, 117.02 ± 17.47 (L) 120.28 ± 17.46 (M) 119.15 ± 8.56 (H),/; GSH-Px/U^**.**^L^-1^:/, 117.02 ± 17.47 (L) 120.28 ± 17.46 (M) 119.15 ± 8.56 (H),/; BLA/ng^.^100mL^-1^: 24.49 ± 1.99,/, 25.41 ± 2.18 (L) 24.54 ± 2.64 (M)23.44 ± 2.56 (H),/; MDA/nmol^**.**^L^-1^ : 14.04 ± 2.07,/, 13.63 ± 2.11 (L) 13.27 ± 1.70 (M) 13.09 ± 1.21 (H),/	
	herpetrione		Swimming time↑, survival time↑; HG/mg^**.**^g^-1^; 9.99 ± 1.58,/, 10.87 ± 1.38(L) 11.67 ± 1.37 (M) 11.75 ± 1.25 (H),/; LDH/U^**.**^L^-1^:874.50 ± 64.22,/,1003.27 ± 92.20 (L) 1046.10 ± 109.91 (M) 1092.73 ± 109.60 (H),/; SOD/U^**.**^mL^-1^: 69.52 ± 9.79,/, 115.18 ± 11.96 (L) 10,220.44 ± 8.07 (M) 123.04 ± 11.36 (H),/; GSH-Px/U^**.**^L^-1^: 109.56 ± 9.58, 109.56 ± 9.58/, 115.18 ± 11.96 (L) 120.44 ± 8.07 (M) 123.04 ± 11.36 (H),/; BLA/ng^.^100mL^-1^: 24.49 ± 1.99,/, 22.40 ± 1.85 (L) 21.75 ± 1.78 (M) 20.91 ± 1.91 (H),/; MDA/nmol^**.**^L^-1^: 14.04 ± 2.07/, 12.15 ± 1.14 (L) 11.65 ± 1.24 (M) 11.50 ± 1.21 (H),/	
Anti-tumor	Lignans	Cell: BEL-7402, BEL-7404, HCT	IC50: 1.45 μg/mL, 1.68 μg/mL, 2.36 μg/mL	Yuan (2006a)
Anti-hyperuricemia	Ethanol	Animal: KM mice (male); Model: Intraperitoneal injection of potassium oxonate emulsion (300 mg/kg). Treatment: ethanol extract (100, 200, 400 mg/kg) for 10 days. Positive control: colchicine (0.3 mg/kg) for 10 days	UA↓, XO(/)	Wang et al. (2022b)
Anti-gouty arthritis			Weight↑, Articular swelling↓, IL-1β↓, TNF-α↓, UA↓	Wang et al. (2022b)
Anti-cholestasis	Ethyl acetate	Animal: SD rats (male); Model: Induction of ANIT (60 mg/kg); Treatment: Ethyl acetate extract (100, 200, 400 mg/kg) for 7 days Positive control: UDCA (100 mg/kg) for 7 days	ALT↓, AST↓, ALP↓, γ-GTP↓, TBIL↓, DBIL↓ TBA↓, GSH↓, SOD↓, GPx↓, CAT↓	Wei et al. (2020)
Anti-skin inflammation	Ethanol	Animal: BALB/c mice (female); Cell: HaCat Model: Mice induced by IMQ for 7 days; Hacat cell induced by IFN-γ (2 ng/mL) for 24 h Treatment: ethanol extract (0.125, 1.25 and 12.5 μg/g) in mice for 7 days. Ethanol extract (12.5 mg/mL) in HaCat cell	IFN-γ↓, TNF-α↓, IL-17A↓, ICAM-1↓, CXCL9↓	Zhong et al. (2023)
Anti-candida albicans	Herpetin, herpetrione	—	Minimal inhibitory concentration of 10.5 μM and 9.2 μM, respectively	Dai et al. (2019)

### 5.1 Hepatoprotective effect

Liver is a vital metabolic organ implementing multiple functions such as toxicant detoxification, protein synthesis, and special compound production, thus the increasing prevalence of liver illnesses including fatty liver, liver damage, fibrosis, cirrhosis, and cancer aroused great attention nowadays (Asrani et al., 2019). As collected in Table 2, plentiful researches showed the remarkable hepatoprotective effect of *H. pedunculosum* seeds through the adjustment of some enzymes in animal models with the induction of CCl_4_, paracetamol (APAP), concanavalin A (ConA), α-naphthyl isothiocyanate (ANIT), liquor, *bacillus* calmette-guérin (BCG) and lipopolysaccharides (LPS). For instance, the ethyl acetate extract of *H. pedunculosum* seeds (EAEHPS) showed hepatoprotective activity against CCl_4_-induced hepatic fibrosis in rats via the inflammatory pathway with obviously inhibiting the expression of NF-κB (IκBα), Samd3, and TGF-β1 proteins (Feng et al., 2018a). The water extract of *H. pedunculosum* seeds could alleviate APAP-induced liver injury by inhibiting oxidative stress and ferroptosis through activating the Nrf2 signal pathway (Li N. Z. et al., 2023). In addition, some proteins, such as NLRP3, TLR-2, TLR-4, and JNK, will have their expression reduced by the total lignan of *H. pedunculosum* seeds (TLHPS), so as to protect mice against ANIT-induced liver damage (Li J. et al., 2023). Some metabolites such as herpetfluorenone (HPF, 23) and herpetin (18) from *H. pedunculosum* seeds were further found to have a positive pharmaceutical effect on acute liver injury by promoting the differentiation of bone marrow mesenchymal stem cells into hepatocellular-like cells and controlling autoimmune oxidation (Yang et al., 2023; Ding et al., 2023).

**FIGURE 7:**
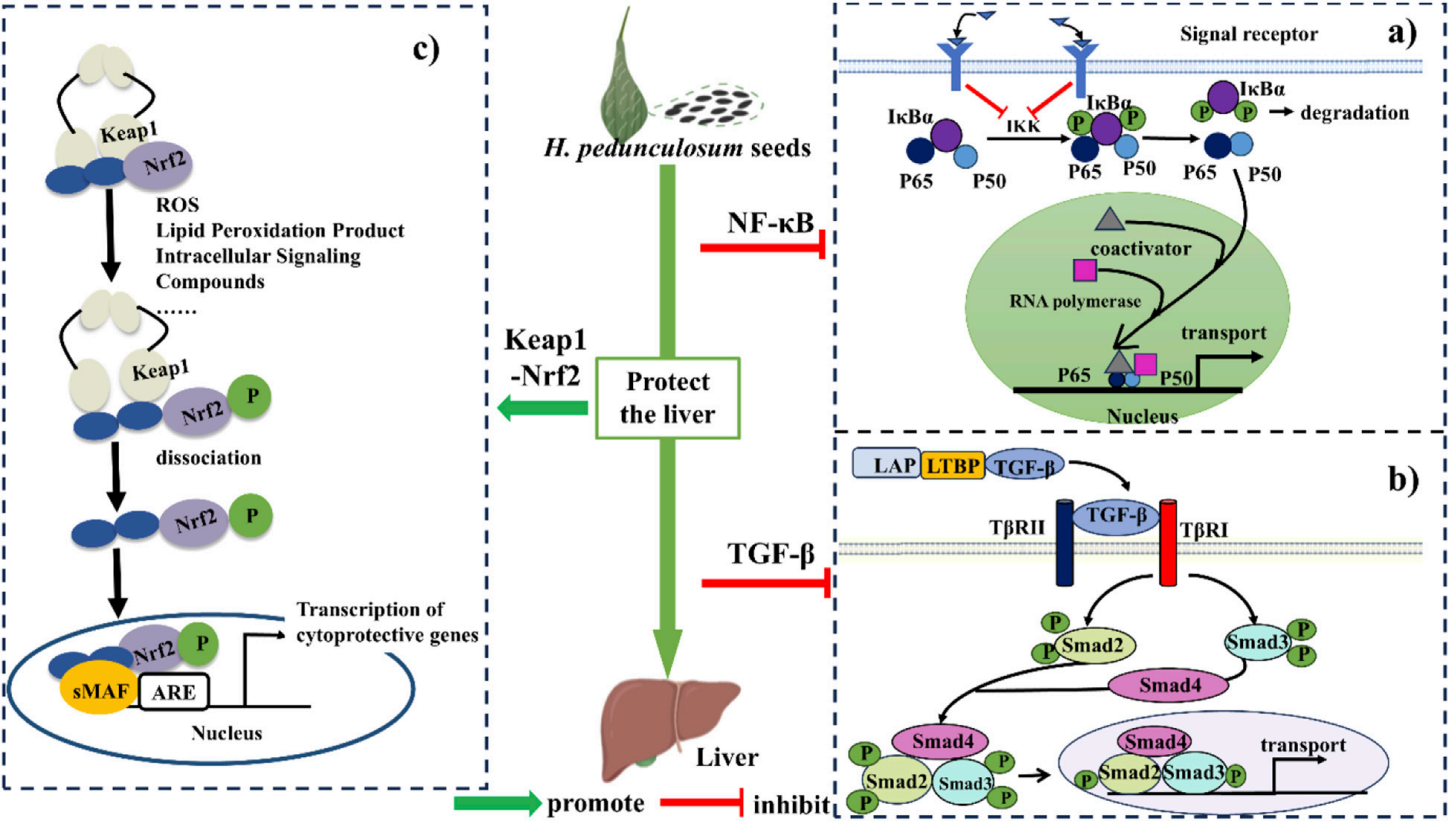
The mechanism of protective effect of *Herpetospermum pedunculosum* seeds on liver. **(A)** NF-κB signaling pathway; **(B)** TGF-β signaling pathway; **(C)** Keap1-Nrf2 signaling pathway.

Based on above discussions and previous literatures, the hepatoprotective mechanism of *H. pedunculosum* seeds can be summarized into three pathways as illustrated in Figure 7. The first one is the inhibition of NF-κB signaling pathway to alleviate the inflammation during liver diseases (Figure 7A). *Herpetospermum pedunculosum* seeds can inhibit the phosphorylation of IκB through the inhibition of IKK, which in turn has anti-inflammatory and hepatoprotective effects (Li N. Z. et al., 2023). The second mechanism is inhibiting the TGF-β signaling pathway (Figure 7B). EAEHPS an inhibit the phosphorylation of Smad3, which in turn inhibits the expression of relevant genes after the complex enters the nucleus, thus playing a role in inhibiting liver fibrosis (Feng et al., 2018a). The third one is the promotion of Keap1-Nrf2 signaling pathway (Figure 7C). Nrf2 plays a crucial role in cellular defense against oxidative stress. When activated by *H. pedunculosum* seeds, the stability of Nrf2 increases, leading to reduced degradation and subsequent activation of genes driven by the antioxidant response element (ARE), thereby exerting a protective effect against liver damage (Li N. Z. et al., 2023; Liao, 2023).

### 5.2 Antioxidation

Fang et al. (2007), Fang et al. (2008) demonstrated the antioxidant activities of CHCl_3_, water and ethanol extracts of *H. pedunculosum* seeds to prevent lipid peroxidation brought on by CCl_4_ *in vivo* experiments. Jiang et al. (2020) tested the significant inhibitory activity of neocucurbitacin D (92) (IC50 = 15.27 ± 0.29 μM) and 23, 24-dihydrocucurbitacin B (97) (IC50 = 24.18 ± 0.26 μM) on XOD. Gong, (2013) showed that herpetone (7) has good DPPH free radical scavenging ability and antioxidant activity. Although many studies have shown that *H. pedunculosum* seeds has antioxidant effects, there are still some problems, such as the simplistic evaluation index and the unclear relationship between dose and activity.

### 5.3 Anti-cancer cells

The lignan of *H. pedunculosum* seeds demonstrated considerable *in vitro* inhibitory action against several cancer cells. The IC50 of lignans in *H. pedunculosum* seeds were 1.45 μg/mL, 1.68 μg/mL, and 2.36 μg/mL for human hepatocellular carcinoma cells (BEL-7402, BEL-7404), and HCT, respectively (Yuan, 2006a). Zhang et al. (2007) demonstrated the inhibitory effects of herpetolide A (102) and herpetolide B (116) on the growth of human promyelocytic leukemia cells (HL-60). The metabolites of *H. pedunculosum* seeds including including herpetosiol A (42), herpetosiol C (44), 7′, 8′-didehydlroherpepetotriol (14), herpetetrol (2), herpepropenal (13), herpetrione (6) showed significant cytotoxicity on human gastric adenocarcinoma cells (SGC7901), human lung cancer cells (A549), human breast cancer cells (MDA-MB-231), and human hepatocellular carcinoma cells (HepG2) (Ma, 2020; Kong et al., 2023). However, these studies only perform a simple detection of IC 50 and cytotoxicity, and lack other powerful indicators to reflect the efficacy of the drug. In addition, it is worth noting that the anti-tumor effects are mainly tested at the cellular level, lacking in animal and mechanism investigations, which are noteworthy in further research.

### 5.4 Anticholestasis effects

EAEHPS exerted an anti-cholestatic effect with increasing bile flow in a dose-dependent manner, which promoted bile acid transport by activating the farnesoid X receptor (FXR) signaling pathway (Wei, 2020). Meanwhile, the EAEHPS activated the Keap1-Nrf2 pathway to alleviate oxidative stress and inhibit of NF-κB/Are signaling pathway to inhibit inflammatory response, which could prevent and treat ANIT-induced cholestasis in rats (Wei et al., 2020).

### 5.5 Other effects

Wang S. W. et al. (2022) found that EAEHPS also had anti-hyperuricemia and anti-gouty arthritis activities, through reducing serum uric acid (UA) levels, suppressing the production and releasing pertinent inflammatory components, and lessening inflammatory damage and pathological tissue necrosis. Jin et al. (2016) demonstrated the anti-fatigue effects of ethanol extract of *H. pedunculosum* seeds with longer swimming time and hypoxia tolerance of experimental mice than that of the control group. The ethanol extract further showed a therapeutic effect on skin inflammation caused by imiquimod (Zhong et al., 2023). Moreover, Dai et al. (2019) showed that herpetin (18) and herpetrione (6) had favorable anti-candida albicans effects with minimal inhibitory connection of 10.5 μM and 9.2 μM, respectively.

## 6 Structure-activity relationship of lignan

Considering the key role of lignans in *H. pedunculosum* seeds, their structure-activity relationship was summarized according to previous literatures. For benzofuran lignans (Figure 2), H-5 can improve its anti-inflammatory capacity when it remains unchanged (Wang L. X. et al., 2022). The electron-withdrawing or electron-donor groups on the benzene ring of benzofuran lignans can decrease their anti-tuberculosis activity (Xu Z. et al., 2019). The anti-tumor activity of tetrahydrofuran lignans with the 7-O-9′ structure (Figure 2B.) can be increased by fixing the following sites: C-7′ is carbonyl group, H-5/5′is not substituted, and C-4/4′is methoxy (Wang L. X. et al., 2022). The antioxidant capacity of furofuran lignans is reported to decrease with the number of substituted methoxy groups on their benzene ring (Wang L. X. et al., 2022). Meanwhile, the presence of methoxy benzene in furofuran lignans enhances its toxicity to tumor cells (Xu W. H. et al., 2019), which could provide a structure-activity basis for the anti-tumor effect of herpetrione (6, Figure 3) (Yuan, 2006a; Yuan et al., 2006b; Yuan et al., 2011). For dibenzylbutane lignans (Figure 2), stronger antiviral activity can be achieved when the hydrogens at C-4 and C-5 are substituted by hydroxyl and methoxy groups respectively, and that at C-3'/4'/5′are substituted by methoxy or hydroxyl groups (Xu et al., 2022). Therefore, the separation and structural modification of lignan compounds from *H. pedunculosum* seeds show great potential for the development of drug leads.

## 7 Pharmaceutical analysis

*Herpetospermum pedunculosum* seeds are only stipulated qualitatively in the Chinese Pharmacopoeia, and their quantitative provisions are still lacking. The existing regulations are not enough to accurately evaluate the quality of *H. pedunculosum* seeds. Therefore, this section briefly introduces the latest research on modern analytical methods to provide guidance for quality evaluation for *H. pedunculosum* seeds.

Lignans such as herpetrione (6), herpetotriol (12), herpetin (18), and herpetfluorenone (23) are considered to be typical metabolites of the genus *Herpetospermum* and also the main active metabolites of *H. pedunculosum* seeds, which undoubtedly have a direct effect on the quality research of *H. pedunculosum* seeds and are indispensable to be detected. Wang, (2014) used herpetotriol (12) as the chemical reference materials in TLC to compare H. pedunculosum seeds from different areas. Cong et al. (2008) detected seven lignans from different areas by reversed-phase HPLC method. The results showed that herpetrione (6) was the most abundant among the seven metabolites, followed by herpetotriol (12). Qian et al. (2011) further accurately analyzed the average content of herpetrione (6) in 10 batches of *H. pedunculosum* seeds from different areas by UPLC, which was found to be 3.7223 mg g^-1^. Except lignan, other metabolites such as fatty acids and polysaccharides also contribute to the bioactivity of *H. pedunculosum* seeds, and their analysis are meaningful for the quality evaluation of *H. pedunculosum* seeds. Ling et al. (2018) detected four fatty acids in *H. pedunculosum* seeds by GC, the result showed that the content of oleic acid (82) was highest, followed by palmitic acid (81). Liu M. L., (2017a) combined UV-Vis and phenol-sulfuric acid methods to detect the polysaccharides in 10 batches of *H. pedunculosum* seeds, indicating higher polysaccharide content (2.16%) of *H. pedunculosum* seeds produced in Yunnan province. However, it is difficult to accurately evaluate the quality of *H. pedunculosum* seeds based on single or several metabolite analyses owing to its plentiful active metabolites, and establishing their fingerprint for similarity evaluation and principal component analysis could be a feasible choice in this aspect. Wang, (2014) found that there were 18 common peaks in the HPLC fingerprint, and the content of herpetolide A (102) was relatively high in all the samples to be analyzed. Subsequently, the HPLC fingerprint of *H. pedunculosum* seeds from Nyingchi region of Tibet was also studied, and 17 common peaks were identified, among which herpetrione (6) was the highest (Chen et al., 2020b).

In brief, among the active metabolites suitable for quantitative analysis, herpetrione (6) and herpetolide (102) exhibit various pharmacological activities with relatively high content, which have the potential to serve as markers for evaluating the quality of *H. pedunculosum* seeds. The current analysis methods for *H. pedunculosum* seeds are still far from perfect to establishing their quality evaluation system. It is urgent to elucidate the key indicative metabolites of *H. pedunculosum* seeds and develop standard determination methods capable of evaluating its quality comprehensively.

## 8 Processing

Processing methods are able to change the effect of *H. pedunculosum* seeds. For example, the stir-frying with grit can effectively alleviate the side effects of diarrhea caused by the shell of *H. pedunculosum* seeds. Meanwhile, the content of herpetrione (6) significantly decreased by 40.9% during this process, which can affect the clinical efficacy (Ling et al., 2018). Research further revealed that *H. pedunculosum* seeds processed by stir-frying with vinegar has better effects on protecting the liver and reducing enzymes, compared with sand owing to the lower herpetrione loss (12%) than that stir-frying with sand (41.4%). (Chen et al., 2016). Additionally, preparing the lignans of *H. pedunculosum* seeds into nanosuspension can improve their bioavailability and stability (Li et al., 2018; Shen et al., 2016). Therefore, the processing optimization could be a feasible approach to enhance the efficacy of *H. pedunculosum* seeds, which deserves more detailed research.

## 9 Application

The commercial herbal formulae including *H. pedunculosum* seeds and related details were collected in Table 4. For example, *H. pedunculosum* seeds is often combined with *Swertia bimaculata*, *Terminalia schedule*, and *Carthami flos* (1, 2, 3 in Table 4) to soothe the liver, promote bile flow, clear heat, and detoxify. When it is paired with *Rosa multiflora*, *T. schedule*, *Phylanthus emblica* (1, 2, 3, 4 in Table 4), formed compound medicines have the effects of strengthening the spleen, as well as promoting digestion. These summarizations and analyses supported the clinical practice of *H. pedunculosum* seeds and provided reference value for the development of other *H. pedunculosum* seed-derived prescriptions.

**TABLE 4:** Formulations and preparations of *H. pedunculosum* seeds.

No	Name	Composition	Efficacy	Refs.
1	Sanwei Qiangwei powder	*Rosa multiflora*, *Herpetospermum caudigerum*, *Terminalia chebula*	Clear heat and remove toxins, promote bile flow, For the treatment of Tri-pa and gallbladder diseases	Chinese Pharmacopoeia Commission (1995)
2	Jiuwei Zhangyacai pill	*Swertia bimaculata*, *Herpetospermum caudigerum*, *Aconitum tanguticum*, *Ixeris polycephala*, *Berberis kansuensis*, *Lagotis Gaertn*, *Hypecoum erectum*, *Radix Aucklandiae*, *Chrysosplenium sinicum*	Clear heat, anti-inflammatory, alleviates pain. For cholecystitis and incipient icteric hepatitis	
3	Wuwei Jinse pill	*Terminalia chebula*, *Herpetospermum caudigerum*, semen punicae granati, faeces soris scrofae, *Radix aucklandiae*	Clear heat and promote bile flow, promote digestion. For the treatment of jaundice hepatitis, and gallbladder pain	
4	Shiwei gaining pill	*Carthami flos, Crocus sativus, Herpetospermum caudigerum, Meconopsis integrifolia, Dracocephalum tanguticum, Saxifraga stolonifera, Corydalis impatiens, Bear gallbladder, Calculus bovis, Brag-zhun, Calciosinti, and Turquoisis*	It is used to treat fatty liver, viral hepatitis, liver fibrosis, cirrhosis, and other liver injuries	Feng et al. (2018b)
5	Qiwei hezi powder	*Terminalia chebula, Herpetospermum caudigerum, Bombax ceiba, Amomum tsaoko Crevost, Syzygium aromaticum, Nardostachys chinensis, Piper longum*	Clear heat and relieve pain. It is used for spleen enlargement, pain and spleen heat caused by strain injury	Yuandan and Song (1987)
6	Songshi pill	Songshi, Borneol*, Syzygium aromaticum, Santalum album,* Pulvis fellis ursi, Forest musk abelmosk*, Herpetospermum caudigerum*	Clear heat and remove toxins, soothe liver. It is used to treat liver pain, cirrhosis, hepatitis and cholecystitis	Xu et al. (2023)
7	Shiwei heibingpian powder	Borneol, *Pomegranate seed, Cinnamomum cassia, Myristica fragrans, Piper longum、Terminalia chebula、Light halititum, Herpetospermum caudigerum、Holarrhena antidysenteriaca,* Pulvis fellis ursi	It is used to treat nausea, cholecystitis, gallstones, and jaundice	Cang and De (2020)

## 10 Conclusions and prospects

*Herpetospermum pedunculosum* seeds is a traditional Tibetan medicine with long history, rich chemical metabolites and high medicinal value. The research of *H. pedunculosum* seeds has achieved fruitful results and provided a scientific basis for the clinical medication. However, there are some shortcomings that need to be addressed in follow-up studies.

Although *H. pedunculosum* seeds is present in many Chinese patent medicines for the treatment of liver diseases, the interaction between the chemical metabolites in the prescriptions remains unclear and needs further investigation. Secondly, the supply of *H. pedunculosum* seeds is restricted due to the particular growth environment and limited wild resources. The large-scale cultivation of *H. pedunculosum* seeds could be of high research and economy value. *Herpetospermum pedunculosum* seeds are reported to contain 125 chemical metabolites, and lignan, terpenoids and coumarin are the main metabolites. Among them, lignan has been widely studied, which is usually recognized as the main pharmacological metabolite of *H. pedunculosum* seeds to exert hepatoprotective effect. However, the research on many other potentially active components such as polysaccharide is still in shortage. More advanced technologies can be used to extract, enrich, separate and purify the metabolites with low content and attention for better understanding of the medicinal material base of *H. pedunculosum* seeds. Moreover, there is a lack of structure-activity relationship studies of other active mmetabolites except lignans in *H. pedunculosum* seeds. The systematic structure-activity relationship studies can accelerate the synthesis of active metabolites and the development of related drugs derived from *H. pedunculosum* seeds.

The pharmacological effects of *H. pedunculosum* seeds, especially its effects on liver diseases, have been extensively researched. However, there are few in-depth studies on other pharmacological effects, and the current pharmacological research only remains at the cell and animal levels without comprehensive clinical research. Future research should take this as the direction to accelerate the clinical translation of drugs. Moreover, the quality standard of *H. pedunculosum* seeds still lacks the indicative components and standard detection method, which can be disadvantageous for standard pharmacology research and clinic practice. At present, some analytical methods have been used to detect the main bioactive ingredients with relatively high content such as herpetrione (6) and herpetolide (102), which may be a promising direction for better quality evaluation.

Although the current medical use of *H. pedunculosum* seeds is without processing, some studies have shown that *H. pedunculosum* seeds stir-fried with sand and vinegar can reduce their side effect of diarrhea and also the content of active ingredients. Therefore, the effect of processing method needs to be systematically determinated and optimized in combination with pharmacology and clinical research. In summary, this paper has comprehensively reviewed and analyzed the botany, phytochemistry, pharmacology, analytical methods and quality evaluation, processing and application of *H. pedunculosum* seeds, which can provide more insights for further research and development of traditional Tibetan medicine.
